# Low-level HIV-1 viremia affects T-cell activation and senescence in long-term treated adults in the INSTI era

**DOI:** 10.1186/s12929-024-01064-z

**Published:** 2024-08-19

**Authors:** Violeta Lara-Aguilar, Manuel Llamas-Adán, Óscar Brochado-Kith, Celia Crespo-Bermejo, Sergio Grande-García, Sonia Arca-Lafuente, Ignacio de los Santos, Carmen Prado, Mario Alía, Coral Sainz-Pinós, Amanda Fernández-Rodríguez, Luz Martín-Carbonero, Ricardo Madrid, Verónica Briz

**Affiliations:** 1 National Center of Microbiology, Institute of Health Carlos III, Madrid, Spain; 2 Centro de Investigación Biomédica en Red de Enfermedades Infecciosas (CIBERINFEC), Institute of Health Carlos III, Madrid, Spain; 3 La Princesa University Hospital, Madrid, Spain; 4 La Paz University Hospital (IdiPAZ), Madrid, Spain; 5 Complutense University, Madrid, Spain; 6 Flow Cytometry Unit, Institute of Health Carlos III, Madrid, Spain

**Keywords:** LLV, HIV-1, Activation, Senescence, Inflammation

## Abstract

**Background:**

Around 10% of people with HIV (PWH) exhibit a low-level viremia (LLV) under antiretroviral therapy (ART). However, its origin and clinical significance are largely unknown, particularly at viremias between 50 and 200 copies/mL and under modern ART based on integrase strand transfer inhibitors (INSTIs). Our aim was to characterize their poor immune response against HIV in comparison to individuals with suppressed viremia (SV) and non-HIV controls (NHC).

**Methods:**

Transversal observational study in 81 matched participants: 27 PWH with LLV, 27 PWH with SV, and 27 NHC. Activation (CD25, HLA-DR, and CD38) and senescence [CD57, PD1, and HAVCR2 (TIM3)] were characterized in peripheral T-cell subsets by spectral flow cytometry. 45 soluble biomarkers of systemic inflammation were evaluated by immunoassays. Differences in cell frequencies and plasma biomarkers among groups were evaluated by a generalized additive model for location, scale, and shape (GAMLSS) and generalized linear model (GLM) respectively, adjusted by age, sex at birth, and ART regimen.

**Results:**

The median age was 53 years and 77.8% were male. Compared to NHC, PWH showed a lower CD4+/CD8+ ratio and increased activation, senescence, and inflammation, highlighting IL-13 in LLV. In addition, LLV showed a downtrend in the frequency of CD8+ naive and effector memory (EM) type 1 compared to SV, along with higher activation and senescence in CD4+ and CD8+ EM and terminally differentiated effector memory RA+ (TEMRA) subpopulations. No significant differences in systemic inflammation were observed between PWH groups.

**Conclusion:**

LLV between 50 and 200 copies/mL leads to reduced cytotoxic activity and T-cell dysfunction that could affect cytokine production, being unable to control and eliminate infected cells. The increase in senescence markers suggests a progressive loss of immunological memory and a reduction in the proliferative capacity of immune cells. This accelerated immune aging could lead to an increased risk of developing future comorbidities. These findings strongly advocate for heightened surveillance of these PWH to promptly identify potential future complications.

**Supplementary Information:**

The online version contains supplementary material available at 10.1186/s12929-024-01064-z.

## Background

According to the latest World Health Organization (WHO) data, there were 40.4 million people with human immunodeficiency virus (HIV) infection in the world in 2023 [1]. In 2022, 29.8 million (76%) people with HIV (PWH) were receiving antiretroviral therapy (ART) and almost 71% of them achieved viral suppression [2]. Currently, American and European guidelines have established virological failure (VF) as any viral load above 50 copies/mL [3, 4]. Between 3 and 23% of PWH on ART show viral loads below 1000 copies/mL [5–7], being the viremias between 200 and 1000 copies/mL consistently associated with VF, HIV drug resistance, and worse clinical outcomes [5–7]. However, the clinical significance of low-level viremia (LLV) below 200 copies/mL remains a matter of ongoing debate, highlighted by the lack of studies in the context of current ART, based on integrase strand transfer inhibitors (INSTIs).

Although treatment has improved the lives of many people, it fails to completely reverse the effects of viral infection on the immune system. HIV causes dysregulation of immune responses, such as increased inflammation and activation, decreased thymic production, and increased conversion of naïve (N) to memory/effector T-cells [8]. This leads to clonal depletion and exhaustion of CD4+ and CD8+ T-cells, which in turn leads to a progressive loss of immunological memory and a reduction in the proliferative capacity of immune cells [9]. Therefore, PWH show accelerated immune aging compared with the general population [9], leading to an increased risk of developing comorbidities such as cancer or cardiovascular diseases [8].

Memory CD4+ and CD8+ T-cells are long-lived populations that provide remarkable cellular protection against infections and malignancies. However, they can also serve as a reservoir for HIV. Very recently it has been described that the proviruses causing LLV are compartmentalized in effector memory (EM) T-cells [10]. In humans EM and terminally differentiated effector memory RA+ (TEMRA) T-cells are frequently defined as CCR7-CD45RA- and CCR7-CD45RA+, respectively. However, several studies have reported heterogeneity within the EM and TEMRA compartments and alternative or complementary approaches for the definition of memory populations based on different levels of CD27 and CD28 have been suggested. In the CD4+ EM T compartment, three subsets have been identified according to the pattern of cytokine production, including a subset that comprises Th0 and Th1 EM cells (Th0-1), another subset predominantly composed of Th1, and a third subset that includes Th1 and Th2 (Th1-2) [11]. For the TEMRA compartment, two subsets have been described: pre-effector 1 (pE1) and effector (E) [12]. For the CD8+ compartment, four EM subpopulations (EM1, EM2, EM3, and EM4) have been distinguished, as well as three TEMRA subsets (pE1, pE2, and E) [12].

So far, studies conducted in PWH with LLV do not discriminate within the heterogeneity of CCR7/CD45RA memory populations [10]. Most investigations that do take this diversity into account focus mainly on healthy donor samples [11, 12] and only a few on PWH but with suppressed viremia [13, 14]. The latter studies show a maturation block leading, for example, to the accumulation of incompletely differentiated CD8+ T-cells (CD27+CD28-). This phenotype has been linked to an ineffective effector response in terms of cytolytic and cytokine-secreting capacity [14–17], a pathogenic strategy used by HIV. The lack of studies delving into these cellular dynamics during LLV makes it difficult to identify the origin of this lack of total suppression and how it affects the immune system. To our knowledge, this is the first study to investigate in depth the levels of activation and senescence in CD4+ and CD8+ memory T-cell compartments, considering the great heterogeneity within these, in the field of LLV and in the context of the INSTIs era.

## Methods

### Study design and participants

A transversal observational cohort study was performed following a case–control strategy matched by age, sex at birth, weight, height, body mass index (BMI), time on ART (if applicable), and time of HIV infection (if applicable) from an initial cohort of 3274 participants who were attended in two tertiary hospitals in Madrid (Spain), La Paz University Hospital and La Princesa University Hospital during 2019–2021. Cases were defined as PWH with LLV described as at least two consecutive viral loads between 50 and 200 copies/mL for the last two years before sample collection (LLV group) (Supplementary Material 1). For each individual with LLV, an HIV control with suppressed viremia (<50 copies/mL) during the last two years before sample collection (SV group) and a non-HIV control (PCR negative and HIV antibodies negative) (NHC group) were selected. Exclusion criteria were pregnancy, individuals below 18 years old, active HCV infection, lack of optimized treatment based on resistance studies, lack of good adherence, clinical evidence of hepatic decompensation, active drug or alcohol addiction, opportunistic infections, and other concomitant diseases such as autoimmune disease, neoplasia, and cardiovascular diseases.

### Biological material

Plasma and PBMCs from participants were isolated by Ficoll density gradient centrifugation (Lymphocyte Separation Media, MP Biomedicals) from peripheral venous blood extracted in EDTA-tubes. Whether red blood cells remained, they were lysed with Red Blood Cell Lysing Buffer Hybri-Max (Sigma-Aldrich). Plasma was stored at −80°C, whereas PBMCs were cryopreserved in liquid nitrogen until use.

### 14-color flow cytometry

To characterize CD4+ and CD8+ memory T-cell subpopulations, the expression of activation and senescence markers was evaluated along the T-cell development in 1.5×10^6^ cryopreserved PBMCs according to the protocol described in Supplementary Material 2, using a panel of fluorescently conjugated antibodies for surface staining, as indicated in Supplementary Material 3. The gating strategies are described in Supplementary Material 4 and Supplementary Material 5.

### Multiplex immunoassays

To characterize systemic inflammation, plasma was clarified by centrifugation at 10,000xg for 10 min at 4°C. 45 molecules were quantified by the Cytokine/Chemokine/Growth Factor 45-Plex Human ProcartaPlex™ Panel 1 (EPXR450-12171–901) with a bead-based multiplex immunoassay (Luminex xMAP Technology) (Thermo Fisher). This panel included cytokines related to the Th1, Th2, Th9, Th17, Th22, and Treg pathways, inflammatory cytokines, chemokines, and growth factors (Supplementary Material 6).

### Statistical analysis

For the descriptive study of the characteristics of participants, continuous variables were summarized as medians and interquartile ranges (IQR), and categorical variables as frequencies and percentages. Significant differences in categorical data were calculated using the chi-squared test or Fisher’s exact test. The Kruskal–Wallis and Mann–Whitney U tests were used to compare continuous variables among independent groups. For the relative quantification of inflammation, the raw fluorescence intensity of biomarkers was used as previously described [18]. On the one hand, given the diverse and heterogeneous phenotypic distribution across the 286 flow cytometry measurements, along with their nonlinear association with age, sex at birth, and ART, we chose to use a semiparametric modeling framework [The Generalized Additive Models for Location, Scale and Shape, (GAMLSS)] to investigate the comparison in the specific flow cytometric readouts contributing to an altered immune response between the three groups of study (LLV vs. NHC, SV vs. NHC, and LLV vs. SV), as previously used in flow cytometry data [19]. As our cellular data takes values in an open unit interval [0, 1] following a beta distribution, we used a zero/one inflated beta distribution for fitting the GAMLSS model. On the other hand, differences for plasma inflammatory biomarkers were assessed using a generalized linear model (GLM) with a gamma distribution (log-link) (LLV vs. NHC, SV vs. NHC, and LLV vs. SV). Despite being matched for age and sex at birth, these variables were included in the model fit together with ART, which was selected by a stepwise algorithm (p-value < 0.15 at each step), according to previous studies [20]. These models provide the arithmetic mean ratio (AMR) of the compared groups and its significance level (*p*-value). *P*-values were corrected by false discovery rate (FDR) using the Benjamin-Hochberg correction, setting a cutoff point of 0.15. The statistical software R (v. 4.2.3) (www.r-project.org) was used for all the statistical analyses.

## Results

### Clinical and epidemiological characterization of participants

A total of 27 PWH with LLV, 27 PWH with SV and 27 NHC were enrolled in this study. No significant differences were observed among the main epidemiological characteristics. Overall, the cohort consisted mostly of Caucasian (95%) males (77.8%), with a mean age [IQR] of 53 years [48.00–58.00] and a BMI of 25.86 [23.82–28.70] (Supplementary Material 7). Biochemical parameters related to the lipid profile and liver function were significantly increased in PWH compared to NHC, except for HDL, which was higher in NHC (Supplementary Material 8).

In relation to the characteristics associated with HIV infection, no differences were observed between LLV and SV (Table 1). Overall, 55% were men who have sex with men (MSM), with a median time of infection and ART initiation of 16 years, the latter being slightly lower in LLV (median 14 years). The predominant type of ART was INSTI-based, representing 74% in LLV and 48% in SV (p<0.001). Participants with LLV were mainly using a bictegravir (BIC)-based INSTI regimen (57.95%), followed by dolutegravir (DTG) (36.80%). All individuals had CD4+ T-cell counts above 500 cells/mm^3^ and together with CD4 nadir T-cells were not statistically different, although they were slightly lower in LLV.

**Table 1:** Epidemiological and clinical characteristics related to HIV infection

	Total	LLV	SV	*p*
**No**	54	27	27	–
**HIV infection (years)**	16.00 [12.00–22.00]	16.00 [10.50–21.50]	16.50 [12.25–24.00]	0.454
**Transmission route [n (%)]**				
MSM	27 (55.10%)	14 (56.00%)	13 (54.20%)	0.999
Heterosexual contact	15 (30.60%)	8 (32.00%)	7 (29.20%)	
IDU	7 (14.30%)	3 (12.00%)	4 (16.70%)	
**Clinical category (CDC) [n (%)]**				
A	28 (60.90%)	12 (48.00%)	16 (76.20%)	0.152
B	6 (13.00%)	4 (16.00%)	2 (9.50%)	
C	12 (26.10%)	9 (36.0%)	3 (14.30%)	
**Time on ART (years)**	16.00 [10.25–21.75]	14.00 [9.50–19.00]	16.00 [11.50–22.00]	0.401
**ART**				
*Classes [n (%)]*				
INSTI + 2NRTI	18 (33.30%)	13 (48.10%)	5 (18.50%)	**<0.001**
INSTI + PI	1 (1.90%)	1 (3.70%)	–	
INSTI + PI + 2NRTI	1 (1.90%)	1 (3.70%)	–	
INSTI + PI + NRTI	2 (3.70%)	2 (7.40%)	–	
INSTI + NRTI	7 (13.00%)	3 (11.10%)	4 (14.80%)	
INSTI + NNRTI	4 (7.40%)	–	4 (14.80%)	
PI + 2NRTI	7 (13.00%)	6 (22.20%)	1 (3.70%)	
PI + NRTI	2 (3.70%)	1 (3.70%)	1 (3.70%)	
NNRTI + 2NRTI	12 (22.20%)	–	12 (44.40%)	
*Regimen [n (%)]*				
Bitherapy	14 (25.90%)	5 (18.50%)	9 (33.30%)	0.352
Tritherapy	40 (74.10%)	22 (81.50%)	18 (66.70%)	
*INSTI regimen [n (%)]*				
BIC-based	16 (50.00%)	11 (57.90%)	5 (38.50%)	
DTG-based	14 (43.80%)	7 (36.80%)	7 (53.80%)	
RAL-based	1 (3.10%)	1 (5.30%)	–	0.431
CAB-LA-based	1 (3.10%)	–	1 (7.70%)	
**Lymphocyte count**				
CD4+ T (cell/mm^3^)	853.00 [599.50–1106.50]	716.50 [514.50–992.50]	1005.00 [673.00–1161.00]	0.104
CD4+ T nadir (cell/mm^3^)	249.00 [156.75–395.25]	231.50 [85.25–376.25]	275.50 [188.00–391.75]	0.174

Statistics: values are expressed as absolute numbers (%) and median [interquartile range]. *P-values* were estimated by Kruskal–Wallis and Mann–Whitney U test for continuous variables and chi-squared or Fisher’s exact test for categorical variables. Statistically significant values are highlighted in bold

*LLV* PWH with low-level viremia (50–200 copies/mL), *SV* PWH with suppressed viremia (<50 copies/mL), *MSM* Men who have sex with men, *IDU* injection drug use, *ART* antiretroviral therapy, *INSTIs* integrase strand-transfer inhibitors, *PIs* protease inhibitors, *NRTIs* nucleoside analogue reverse transcriptase inhibitors, *NNRTIs* non-nucleoside reverse transcriptase inhibitors, *BIC* bictegravir, *DTG* dolutegravir, *RAL* raltegravir, *CAB-LA* long-acting injectable cabotegravir

### Comparison of immunophenotypic characterization of T-cells between PWH groups (LLV vs SV) with NHC

#### CD4+ T-cell profile

**Fig. 1:**
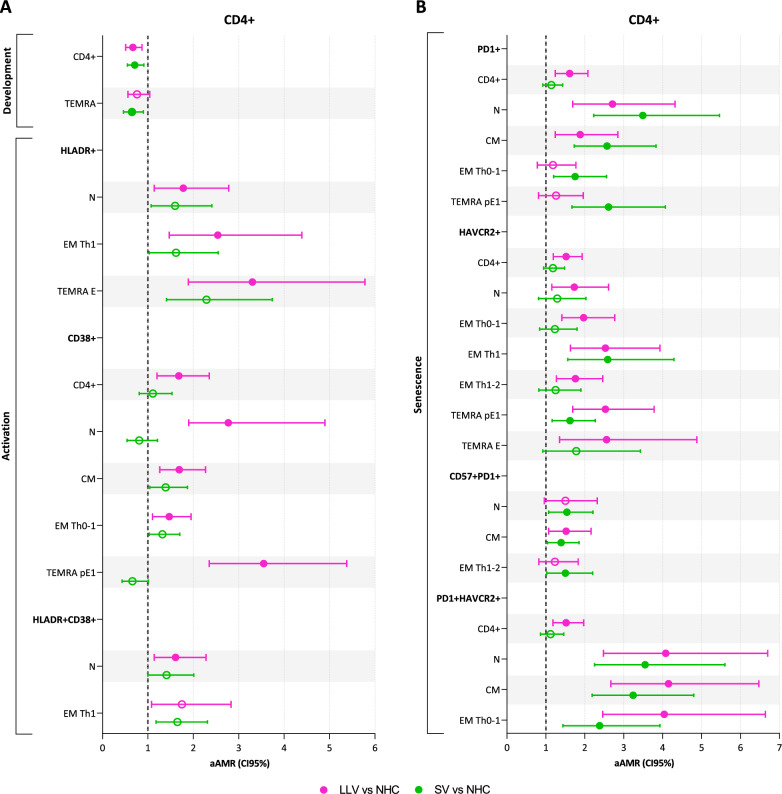
Comparison of CD4+ T-cell profile between both PWH groups with NHC group in relation to **A**: development and activation and **B**: senescence**.** Statistics: The AMR values were obtained using a GAMLSS with beta distribution adjusted by age, sex at birth, and antiretroviral therapy. Only statistically significant results for any of the two comparisons are shown (filled balls, p<0.05 and q<0.15). *LLV* PWH with low-level viremia (50–200 copies/mL), *SV* PWH with virologic suppression (<50 copies/mL), *NHC* non-infected HIV controls, *aAMR* adjusted arithmetic mean ratio, *N* naïve T-cells, *CM* central memory T-cells, *EM* memory effector T-cells, *TEMRA pE1* pre-terminally differentiated effector memory RA+ 1 T-cells, *TEMRA* *pE2* pre-terminally differentiated effector memory RA+ 2 T-cells, *TEMRA* *E* terminally differentiated effector memory RA+ T-cells, HAVCR2 also known as TIM3

PWH groups exhibit a decrease in CD4+ T-cells counts compared to NHC, being significantly more activated CD38+ (Fig. 1A and Supplementary Material 9) and senescent due to the up-regulation of PD1, HAVCR2 (TIM3), and the co-expression of PD1 and HAVCR2 in LLV (Fig. 1B and Supplementary Material 9). A reduction in the frequency of TEMRA cells was also observed in PWH groups, although it was only statistically significant in SV (Fig. 1A and Supplementary Material 9).

No statistically significant differences were found in the intermediate activation phenotype (CD25+HLADR+) (data not shown), but increased activation (HLADR+ and CD38+) was observed throughout the development of CD4+ T-cells in PWH groups compared to NHC, from N to central memory (CM) and several subpopulations of the EM (Th0-1 and Th1) and TEMRA (pE1 and E) responses (Fig. 1A and Supplementary Material 9). However, such higher activation was only statistically significant in LLV, reaching higher levels, and showing a higher frequency of N with a late activation phenotype (HLADR+CD38+) and an upward tendency in the EM Th1 subpopulation (p=0.027, q=0.192) (Fig. 1A and Supplementary Material 9).

LLV showed a significant overall increase in senescence (PD1+ and HAVCR2+) of CD4+ T-cell compared to NHC (Fig. 1B and Supplementary Material 9). However, PD1+ populations were enriched in SV, particularly N, CM, Th0-1, and TEMRA pE1, in contrast to the significantly greater frequency of HAVCR2+ populations observed in LLV, from N to EM (Th0-1, Th1, and Th1-2) and TEMRA (pE1 and E) responses. Finally, while SV exhibited a markedly increase in N, CM, and EM Th1-2 subpopulations with an intermediate senescence phenotype (CD57+PD1+) compared to NHC, LLV showed a remarkable increase in N, CM, and EM Th0-1 subpopulations with an advanced senescence phenotype (PD1+HAVCR2+).

#### CD8 + T-cell profile

**Fig. 2:**
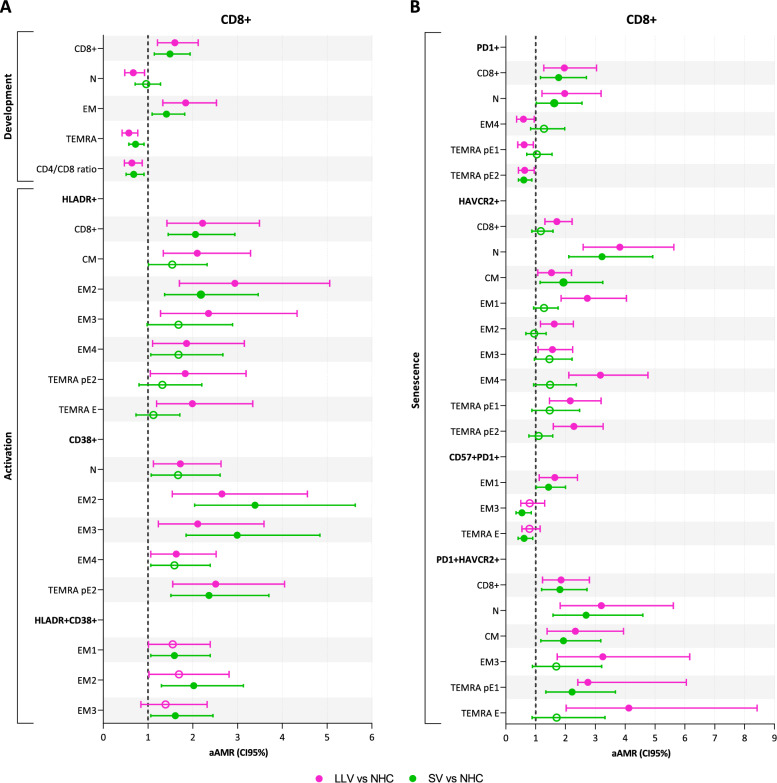
Comparison of CD8+ T-cell profile between both PWH groups with NHC group in relation to **A**: development and activation and **B**: senescence. Statistics: The AMR values were obtained using a GAMLSS with beta distribution adjusted by age, sex at birth, and antiretroviral therapy. Only statistically significant results are represented (filled balls, p<0.05 and q<0.15). *LLV* PWH with low-level viremia (50–200 copies/mL), *SV* PWH with virologic suppression (<50 copies/mL), *aAMR*, adjusted arithmetic mean ratio, *N* naïve T-cells, *CM* central memory T-cells, *EM* memory effector T-cells, *TEMRA pE1* pre-terminally differentiated effector memory RA+ 1 T-cells, *TEMRA* *pE2* pre-terminally differentiated effector memory RA+ 2 T-cells, *TEMRA E* terminally differentiated effector memory RA+ T-cells, *HAVCR2* also known as TIM3

Overall, PWH groups showed an increased frequency of CD8+ T-cells compared to NHC, with higher levels of activation (HLADR+ and CD38+) (Fig. 2A and Supplementary Material 10) and senescence (PD1+, HAVCR2+, and PD1+HAVCR2+) (Fig. 2B and Supplementary Material 10), being higher in LLV. A reduced CD4+/CD8+ T-cell ratio, increased conversion of N to EM T-cells, and decreased frequency of TEMRA were also observed, being a more noticeable alteration in LLV (Fig. 1A and Supplementary Material 10).

No differences were found in the intermediate activation phenotype (CD25+HLADR+) (data not shown), but increased expression of HLA-DR and CD38 markers was observed throughout the effector response (EM2, EM3, and TEMRA pE2). These levels were greater in LLV, which also showed significantly greater activation in N, CM, EM3, EM4, and TEMRA E than in NHC (Fig. 2A and Supplementary Material 10). An increase in EM subpopulations with an advanced activation phenotype (HLADR+CD38+) was also observed, although this elevation was only statistically significant in SV (EM1, EM2, and EM3).

Regarding senescence levels, an increase of PD1 in the absolute number of CD8+ T-cells was observed in PWH groups compared to NHC, as well as an increase of HAVCR2 expression in LLV (Fig. 2B and Supplementary Material 10). LLV also showed a reduction in PD1+ subpopulations (EM4, TEMRA pE1, and pE2) and an increase in HAVCR2 expression throughout CD8+ T development (N, CM, EM1, EM2, EM3, EM4, TEMRA pE1, and pE2). In contrast, SV only exhibits a significant increase in HAVCR2 expression in N and CM compared to NHC. Finally, SV showed a lower proportion of EM3 and TEMRA E subpopulations with an intermediate senescence phenotype. On the other hand, LLV presented a strong increase in advanced senescence levels in different CD8+ T subpopulations (N, CM, EM3, TEMRA pE1, and E), which were also altered in SV, although to a lesser extent, except for EM3 and TEMRA E.

### Comparison of immunophenotypic characterization of T-cells between LLV and SV

#### CD4+ T-cell profile

**Fig. 3:**
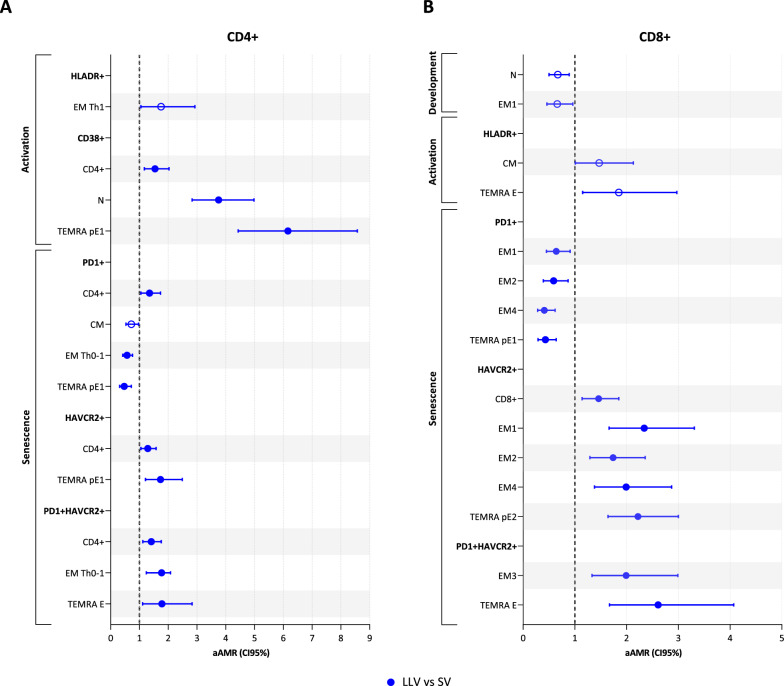
Immunophenotypic profile of **A**: CD4+ T-cells and **B**: CD8+ T-cells in LLV group compared with SV group. Statistics: The AMR values were obtained using a GAMLSS with beta distribution adjusted by age, sex at birth, and antiretroviral therapy. Only statistically significant results are represented (filled balls, p<0.05 and q<0.15). *LLV*, PWH with low-level viremia (50–200 copies/mL), *SV* PWH with virologic suppression (<50 copies/mL), *aAMR* adjusted arithmetic mean ratio, *N* naïve T-cells, *CM* central memory T-cells, *EM* memory effector T-cells, *TEMRA* *pE1* pre-terminally differentiated effector memory RA+ 1 T-cells, *TEMRA pE2* pre-terminally differentiated effector memory RA+ 2 T-cells, *TEMRA E* terminally differentiated effector memory RA+ T-cells, *HAVCR2* also known as TIM3

No significant difference within the CD4+ T-cell development was observed in LLV. However, they did exhibit significantly higher levels of activated (CD38+) and senescent (PD1+, HAVCR2+, and PD1+HAVCR2+) CD4+ T-cells compared with SV (Fig. 3 and Supplementary Material 10).

In particular, the greatest differences were observed at the most immature stages within each developmental stage. LLV showed higher activation of N and TEMRA pE1 cells, as well as a positive trend in EM Th1 cells (p=0.037, q=0.590). A lower frequency of more immature subpopulations expressing PD1+ was also observed within the effector response (EM Th0-1 and TEMRA pE1), as well as a negative trend in CM cells (p=0.036, q=0.159). In addition, an up-regulation of HAVCR2 in TEMRA pE1 was also observed. Finally, no significant differences were observed in the intermediate senescence phenotype but were found in the advanced senescence phenotype (PD1+HAVCR2+), particularly in the EM Th0-1 and TEMRA E subpopulations.

#### CD8+ T-cell profile

The frequency of CD8+ N (p=0.034, q=0.285) and EM1 T-cells (p=0.034, q=0.285) tended to decrease in LLV compared to SV (Fig. 3B and Supplementary Material 10). Similar activation levels were observed throughout CD8+ T-cell development, except for an increasing trend in activated (HLADR+) CM (p=0.048, q=0.993) and TEMRA E (p=0.015, q=0.736) subpopulations in LLV.

In relation to the immune senescence profile, LLV showed a significantly lower frequency of several EM (EM1, EM2, and EM4) and TEMRA pE1 subpopulations expressing PD1+. An up-regulation of HAVCR2 was also observed in CD8+ T-cells in LLV, especially within the EM (EM1, EM2, and EM4) subset and the TEMRA pE2 subpopulation. Finally, no significant differences were found in the intermediate senescence phenotype, but an increase in EM3 and TEMRA E populations with an advanced senescence phenotype was observed.

### Quantification of systemic inflammation

**Fig. 4:**
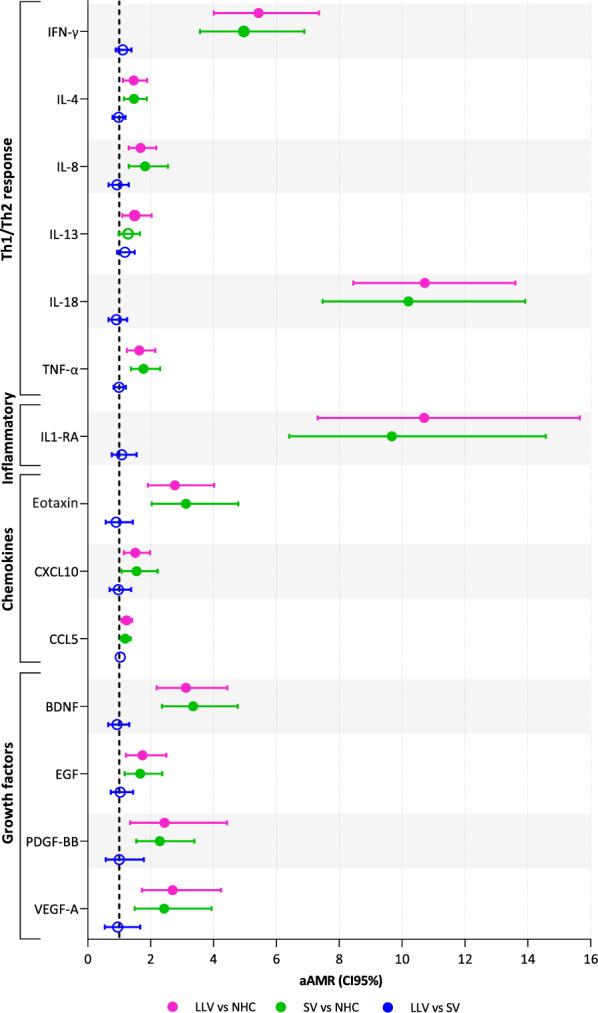
Comparison of the levels of systemic inflammation among the three groups. Statistics: The AMR values were obtained using a GLM with gamma distribution adjusted by age, sex at birth, and antiretroviral therapy. Only statistically significant results for any of the three comparisons are represented (filled balls, p<0.05 and q<0.15). *LLV* PWH with low-level viremia (50–200 copies/mL), *SV* PWH with virologic suppression (<50 copies/mL), *NHC* non-infected HIV controls, *aAMR* adjusted arithmetic mean ratio

In relation to inflammation, PWH groups showed a significant increase in the levels of several cytokines related to the Th1 and Th2 pathways (IFN-γ, IL-4, IL-8, IL-18, and TNF-α); the inflammatory cytokine IL1-RA; the chemokines eotaxin (CCL11), CXCL10 (IP-10), and CCL5 (Rantes); and growth factors (BDNF, EGF, PDGF-BB, and VEGF-A) (Fig. 4 and Supplementary Material 11). A significant increase in IL-13 was found only in LLV compared to NHC. Finally, no statistically significant differences in inflammatory markers were observed between PWH groups (Fig. 4 and Supplementary Material 11).

## Discussion

To the best of our knowledge, this is the first study to deeply explore plasma and cellular markers in CD4+ and CD8+ T-cell pools in a well-characterized cohort of adults over 50 years of age with LLV below 200 copies/mL under long-term ART, mostly based on INSTIs. Our data show that LLV impairs T-cell development and increases cell activation and senescence, possibly leading to an aberrant immune function, which may explain the absence of significant differences in systemic inflammation levels between individuals with LLV and SV.

HIV replicates in the T-cell pool, causing progressive immune system failure [21, 22]. Increased antigenic and inflammatory loads during infection led to increased immune activation and exhaustion in PWH groups compared to NHC, especially in LLV. Blockade of the senescence markers PD1 and HAVCR2 (TIM3) has been shown to potentiate the HIV-1-specific T-cell response [23], which would imply an unbalanced cytotoxic response especially pronounced in LLV compared to NHC.

To date, most of the studies have been performed in virologically suppressed adults or before the introduction of INSTIs [24–26]. Therefore, much is still unknown about the origin of LLV and how it affects the immune system, especially in viremias below 200 copies/mL. Our data showed no differences in CD4+ T-cell development between LLV and SV groups, in agreement with the findings of Han and colleagues (2020), who carried out a study in young adults with LLV [27]. In contrast, we observed increased activation in the CD4+ T pool in LLV, especially in N cells and the more immature subpopulations within effector memory cells (EM Th1 and TEMRA pE1), suggesting that T-cell activation could lead to strong effector functions.

However, such cellular activation was accompanied by a generalized increase in PD1 expression in CD4+ T-cells in LLV, as well as HAVCR2+ throughout CD4+ T-cell development, highlighting an increased senescence phenotype (PD1+HAVCR2+) in the effector memory response (EM Th0-1 and TEMRA E). The up-regulation of HAVCR2 and PD1 has previously been correlated with HIV viral load and a lower absolute CD4+ T-cell count [28–30]. This phenomenon is in line with the observation of the generalized increase in these two markers in LLV, which also showed a more marked decrease in CD4+ T-cells, as mentioned above. The expression of these two exhaustion markers has also been associated with CD4+ and CD8+ T-cell dysfunction via the inhibition of cell proliferation in response to antigens, and the net production of cytokines [28–30].

The up-regulation of PD1 during HIV infection has been extensively documented before, with the highest levels found in HIV-specific T-cells. However, our research revealed a reduced frequency of CM along with immature stages of effector memory subpopulations (EM Th0-1 and TEMRA pE1) expressing PD1 in LLV. Early differentiated PD1+ CD4+ T-cells could constitute a highly functional population with increased susceptibility to HIV infection, leading to selective depletion in chronic infection [31]. The decrease in these populations indicates a potential inability to execute an effective CD4+ T response in LLV, which may partly explain the absence of significant differences in inflammatory biomarkers with respect to SV.

Regarding CD8+ T-cells, LLV showed a lower frequency of CD8+ EM1 compared to SV. To our knowledge, this is the first time this population has been described in the HIV field. A decrease in this subpopulation has been reported in different viral infections, such as during the first week of SARS-CoV-2 infection, when the viral load reaches its highest value [32]. In addition, lower frequencies of CD8+ EM1 T-cells have also been described in individuals with melanoma [33], which seems to have clinical significance because higher levels of this population in the peripheral blood predict a good outcome [34]. In this regard, the clinical relevance of CD8+ EM1 T-cells in LLV remains to be determined, but they are known to express high levels of granzyme K, which targets viral proteins crucial for replication [35], as well as promoting cell apoptosis. This may be especially relevant in the case of HIV-infected cells, where it may contribute to viral clearance. Thus, a decrease in CD8+ EM1 T-cells during LLV could contribute to reduced cytotoxic activity, allowing further spread of the virus.

In addition, LLV also showed a trend toward increased activation of CD8+ TEMRA E T-cells and increased expression of exhaustion markers, primarily within the CD8+ EM and TEMRA T response. The expression of HLA-DR and CD38 on the surface of CD8+ T-cells has previously been correlated with viral loads between 40 and 999 copies/mL. Our results show that even lower viremias, below 200 copies/mL, can leave an unfavorable immunological footprint, with significant consequences for people aged 50 years and above, since the immune systems of people 50 and older tend to recover more slowly compared with those of younger people [36]. Indeed, strong immune activation, elevated levels of circulating viral antigens, and T-cell imbalance may contribute to the development of T-cell exhaustion [21, 22], supporting the widespread up-regulation of HAVCR2 observed in LLV. The upregulation of HAVCR2 in HIV-specific CD8+ T-cells has been associated with reduced effector functions in HIV progressors [28]. In fact, immune exhaustion caused by increased PD1 and HAVCR2 expression in T-cells has been shown to lead to clonal elimination of antigen-specific T-cells, which are unable to control and eliminate infected cells [37]. In addition, very recently it has been described that proviruses causing LLV exhibit varying degrees of escape to cytotoxic T lymphocytes and humoral response, being more pronounced in persistent viremias than in intermittent ones [10].

Therefore, our results suggest that although there is a large majority of activated EM and TEMRA populations in LLV group, most of them may already be functionally exhausted. This could explain why the inflammation was greater in PWH groups than in NHC, but no noteworthy difference was observed between them. Increased inflammation in PWH has been previously observed, being associated with HIV persistence and disease progression, even after effective long-term treatment [38]. The clinical significance of higher IL-13 levels in LLV compared to NHC remains uncertain. Some studies have associated it with positive effects on antigen presentation and the inhibition of HIV-1 infection [39], while others have associated it with cancer, among others [40]. The observed lack of association between viremia below 200 copies/mL and inflammatory markers, such as IL-6, C-reactive protein (CRP), sCD14, CXCL10, Vascular cell adhesion molecule 1 (VCAM-1), and fibrinogen, has been previously demonstrated [41] and we hypothesize that may be due to T-cell exhaustion observed in LLV group. Nevertheless, it is also possible that their production could be triggered solely by high viral loads (above 400 copies/mL), as demonstrated previously [41].

T-cell exhaustion, characterized by increased PD1 and HAVCR2 expression, has also been previously linked to the tumor microenvironment. The expression of these markers in a wide range of malignancies has been a trending topic in recent years, as they appear to have the potential to serve as prognostic markers and valuable therapeutic targets in cancer [42, 43]. In fact, they are currently of great clinical interest due to their demonstrated efficacy in immunotherapy [44, 45]. The correlation between increased expression of these inhibitory coreceptors and cancer is associated with progressive deterioration of the ability of T cells to respond to polyclonal activation and disease progression [46]. Chronic antigen exposure and the development of dysfunctional or exhausted effector T-cells are common features shared by chronic infections such as HIV and cancer. This may partly explain why PWH have a higher incidence of non-AIDS-associated cancers [47, 48], which is also accentuated with increasing age [47]. Thus, the accentuated increase observed in the T-cells of participants with LLV might suggest an increased risk of accelerated onset of neoplastic events.

Finally, it is worth noting the high percentage of participants with LLV using an INSTI-based regimen. This type of therapy has become the mainstay of modern ART [49–51] especially for the management of LLV and subsequent virological failure [52]. This is mainly because they have greater efficacy for virologic control, high genetic barrier, better tolerability, and lower potential for drug-drug interactions than NNRTIs or PIs [53–57]. The presence of LLV even under this type of treatment in our cohort coincides with previous studies observing that 25.3% and 11.4% of participants showed LLV (50–500 copies/mL) after a 48 and 96-week follow-up under INSTIs respectively [58]. However, the impact of this viremia on the immune system of individuals was not studied, so the findings of our study yield new and valuable information in the era of modern HIV treatment. The median treatment time under INSTI in our LLV participants (50–200 copies/mL) was 52 weeks, so it is possible that a longer time under this ART may be necessary to achieve a sustained undetectable viral load. However, whether viral load suppression would allow restoration of the observed immunologic footprint is not yet clear.

To accurately interpret our findings, it is essential to acknowledge certain limitations. Although the sample size was limited, the strengths of this study lie in the use of a well-characterized and matched cohort. In addition, the use of a 14-color multiparametric panel analyzed by spectral flow cytometry that deeply characterizes different T lymphocyte subpopulations, together with appropriate statistical models adjusted for clinical and epidemiological covariates, improves the robustness of our results. Nevertheless, we are unable to determine whether immune alterations observed are the origin or the consequence of the LLV. Further investigations to explore the long-term evolution of T-cell dynamics and inflammatory markers would be an intriguing avenue for future research.

## Conclusion

The persistence of LLV between 50 and 200 copies/mL led to a decrease in CD8+ EM1 and increased activation, mainly in EM and TEMRA populations that could be functionally exhausted. These findings suggest a reduced cytotoxic activity and T-cell dysfunction affecting the cytokine production, which partially explains the absence of significant differences in the inflammatory profile between LLV and SV. Taken together, these findings indicate an aberrant immune function in LLV individuals who are unable to control and eliminate infected cells. These findings strongly advocate for heightened surveillance of these PWH to promptly identify potential future complications, such as adverse clinical outcomes or virological failure.

### Supplementary Information

Supplementary Material 1. Includes Supplementary Material 1-11.

## Data Availability

The material will be available from the corresponding author on reasonable request.

## Abbreviations

aAMR: Adjusted arithmetic mean ratio
AMR: Arithmetic mean ratio
ART: Antiretroviral therapy
BDNF: Brain-derived neurotrophic factor
BIC: Bictegravir
BMI: Body mass index
CCL11: C–C motif chemokine 11
CCL5: CC-chemokine ligand 5
CM: Central memory
CRP: C-reactive protein
CXCL10: C-X-C motif chemokine 10
DTG: Dolutegravir
E: Effector
EDTA: Ethylenediamine tetraacetic acid
EGF: Epidermal growth factor
EM: Effector memory
FDR: False discovery rate
GALMSS: Generalized additive models for location, scale, and shape
GLMs: Generalized linear models
HAVCR2: Hepatitis A virus cellular receptor 2
HDL: High-density lipoprotein
HIV: Human immunodeficiency virus
IFN-γ: Interferon-gamma
IL-13: Interleukin 13
IL-18: Interleukin 13¡8
IL1-RA: Interleukin-1 receptor antagonist
IL-4: Interleukin 4
IL-8: Interleukin 8
INSTIs: Integrase strand transfer inhibitors
IQR: Interquartile range
LLV: Low-level viremia
MSM: Were men who have sex with men
N: Naïve
NHC: Non-HIV control
PBMCs: Peripheral blood mononuclear cells
PD1: Programmed Cell Death Protein 1
PDGF-BB: Platelet-derived growth factor-BB
pE: Pre-effector
PWH: People with HIV
SARS-CoV-2: Severe acute respiratory syndrome coronavirus 2
SV: Suppressed viremia
TEMRA: Terminally differentiated effector memory RA +
TNF-α: Tumor Necrosis Factor-alpha
VCAM-1: Vascular cell adhesion molecule 1
VEGF-A: Vascular endothelial growth factor A
VF: Virological failure
WHO: World Health Organization
