# Supplementary material for: Low-level HIV-1 viremia affects T-cell activation and senescence in long-term treated adults in the INSTI era
Source: J Biomed Sci. 2024 Aug 19;31:80. doi: 10.1186/s12929-024-01064-z (PMC11334306; doi:10.1186/s12929-024-01064-z)
Supplement: Supplementary file 1 — Supplementary Material 1. Includes Supplementary Material 1-11. [file 12929_2024_1064_MOESM1_ESM.docx]

| **Supplementary Material 1.** Flow-chart of participant´s selection. | 1 |
| --- | --- |
| **Supplementary Material 2.** Protocol of 14-color flow cytometry | 1 |
| **Supplementary Material 3.** Fluorescently conjugated antibody panel. | 2 |
| **Supplementary Material 4**. Immunophenotypic characterization of subpopulation CD4+ and CD8+ T cells. | 2 |
| **Supplementary Material 5**. Gating strategy. | 3 |
| **Supplementary Material 6**. Markers of systemic inflammation evaluated in plasma of participants. | 5 |
| **Supplementary Material 7**. Epidemiological characteristics of all group of patients enrolled in this study. | 6 |
| **Supplementary Material 8**. Biochemical and metabolic characteristics of all group of patients enrolled in this study. | 7 |
| **Supplementary Material 9**. Comparison of T-cell development, activation, and senescence levels of CD4+ T-cells between all groups of study. | 8 |
| **Supplementary Material 10**. Comparison of T-cell development, activation, and senescence levels of CD8+ T-cells between all groups of study. | 9 |
| **Supplementary Material 11**. Comparison of systemic inflammation between all groups of study. | 11 |

# **APPENDIX**

# **Supplementary Material 1. Flow-chart of participant´s selection.**


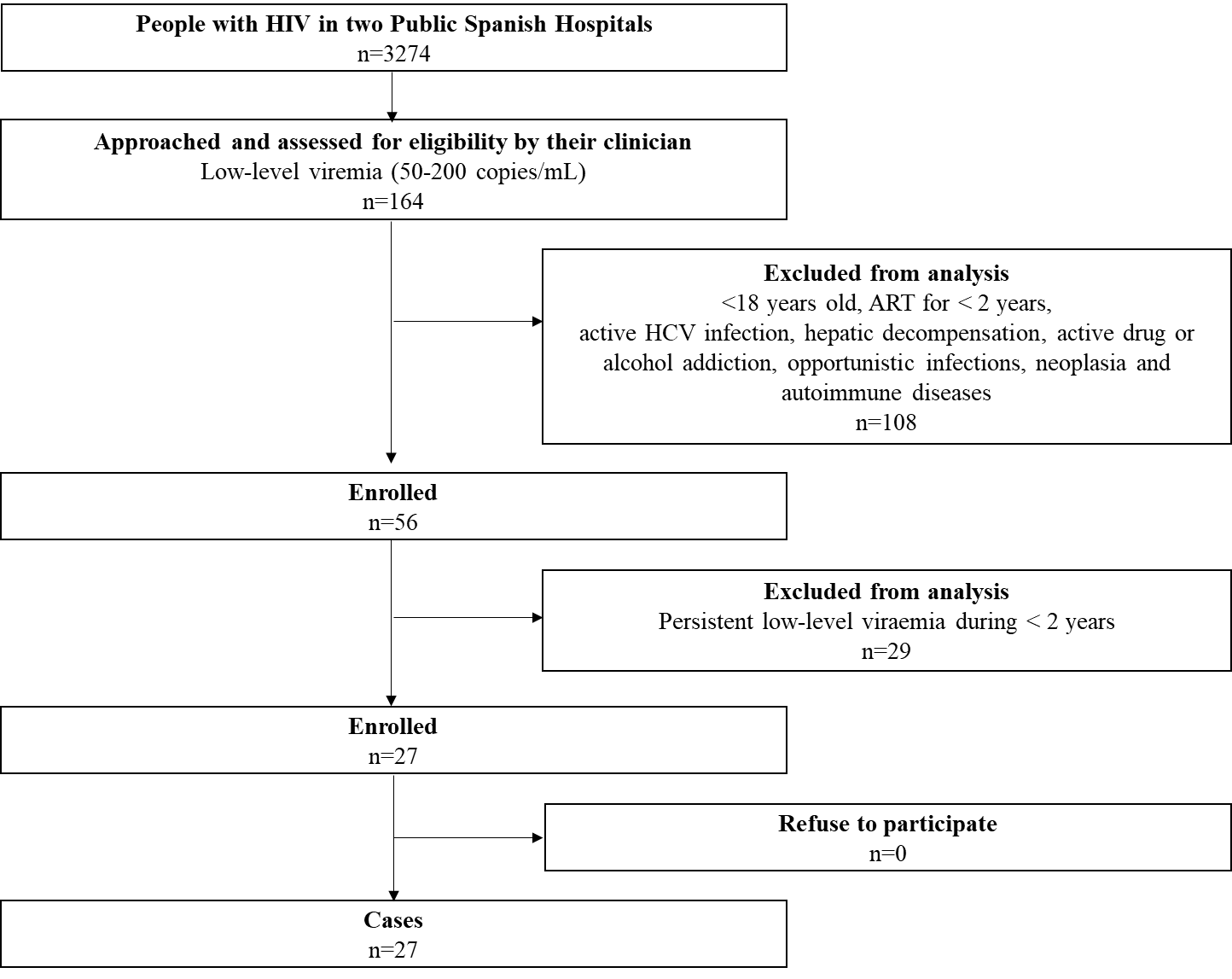


**Notes:** Cases were defined as people with HIV with low-level viremia described as at least two consecutive viral loads between 50-200 copies/mL) for the last two years before sample collection (LLV group). For each case, an HIV control with suppressed viremia (<50 copies/mL) during the last two years (SV group) and a non-HIV control (PCR negative, HIV antibodies negative) (NHC group) were selected. Exclusion criteria were pregnancy, individuals below 18 years old, active HCV infection, lack of optimized treatment based on resistance studies, lack of good adherence, clinical evidence of hepatic decompensation, active drug or alcohol addiction, opportunistic infections, and other concomitant diseases such as autoimmune disease, and neoplasia.

# **Supplementary Material 2. Protocol of 14-color flow cytometry**

To characterize CD4+ and CD8+ T-cells subpopulations, the expression of surface markers was evaluated in 1.5x10^6^ cryopreserved PBMCs stained with Fixable Viability Dye eFluor 455UV (ThermoFisher) 30 min at 4°C. After washing with PBS+2%FBS, non-specific antibody binding was blocked using the Human TruStain FcX (BioLegend) for 10 min at room temperature. After surface staining for 30 min at 4°C and washing, cells were finally fixed with 2% PFA for analysis on an Aurora Spectral flow cytometer with five lasers (Cytek Biosciences). Data were analysed with FlowJo^TM^ v10 software (Tree Star, Ashland).

# **Supplementary Material 3. Fluorescently conjugated antibody panel**

| **Markers** | **Antibodies** | **Reference** |
| --- | --- | --- |
| ***Viability*** | Fixable Viability Dye eFluor 455UV | ThermoFisher |
| ***T-cell development*** | CD3-BUV805 | Beckton-Dickinson |
|  | CD4-BUV395 | Beckton-Dickinson |
|  | CD8-BV510 | Beckton-Dickinson |
|  | CCR7-BUV563 | Beckton-Dickinson |
|  | CD45RA-APC/H7 | Beckton-Dickinson |
|  | CD27-BD421 | Beckton-Dickinson |
|  | CD28-PE/Cy7 | Beckton-Dickinson |
| ***Activation*** | CD25-BV711 | BioLegend |
|  | HLA-DR-AF647 | BioLegend |
|  | CD38-cFLuor R720 | BioLegend |
| ***Senescence*** | CD57-PE/Dazzle 594 | BioLegend |
|  | PD1-BB700 | Beckton-Dickinson |
|  | TIM-3-BV785 | BioLegend |

# **Supplementary Material 4. Immunophenotypic characterization of subpopulation CD4+ and CD8+ T cells.**

| **Population** | **Phenotypic** | **Ref** |
| --- | --- | --- |
| ***CD4+*** |  |  |
| Naïve (N) | CD3+, CD4+, CD45RA+, CCR7+, CD27+, CD28+ | 1,2,3,4 |
| Central memory (CM) | CD3+, CD4+, CD45RA-, CCR7+, CD27+, CD28+ | 1,2,3,4 |
| Effector memory (EM) | CD3+, CD4+, CD45RA-, CCR7- | 1,2,3,4. |
| Effector memory 0 and 1 (EM Th0-1) | CD3+, CD4+, CD45RA-, CCR7-, CD28+, CD27+ | 4 |
| Effector memory 1 (EM Th1) | CD3+, CD4+, CD45RA-, CCR7-, CD28-, CD27- | 4 |
| Effector memory 1 and 2 (EM Th1-2) | CD3+, CD4+, CD45RA-, CCR7-, CD28+, CD27- | 4 |
| Terminally differentiated effector memory RA+ (TEMRA) | CD3+, CD4+, CD45RA+, CCR7- | 1,5 |
| Pre-terminally differentiated effector memory RA+ 1 (pE1) | CD3+, CD4+, CD45RA+, CCR7-, CD28+, CD27+ | 1,5 |
| Terminally differentiated effector memory RA+ effector (E) | CD3+, CD4+, CD45RA+, CCR7-, CD28-, CD27- | 1,5 |
| ***CD8+*** |  |  |
| Naïve (N) | CD3+, CD8+, CD45RA+, CCR7+, CD27+, CD28+ | 1,2,3,4,5 |
| Central memory (CM) | CD3+, CD8+, CD45RA-, CCR7+, CD27+, CD28+ | 1,2,3,4,5 |
| Effector memory (EM) | CD3+, CD8+, CD45RA-, CCR7- | 1,2,3,4,5 |
| Effector memory type 1 (EM1) | CD3+, CD8+, CD45RA-, CCR7-, CD27+, CD28+ | 1,5,6 |
| Effector memory type 2 (EM2) | CD3+, CD8+, CD45RA-, CCR7-, CD27+, CD28- | 1,5,6 |
| Effector memory type 3 (EM3) | CD3+, CD8+, CD45RA-, CCR7-, CD27-, CD28- | 1,5,6 |
| Effector memory type 4 (EM4) | CD3+, CD8+, CD45RA-, CCR7-, CD27-, CD28+ | 1,5,6 |
| Terminally differentiated effector memory RA+ (TEMRA) | CD3+, CD8+, CD45RA+, CCR7- | 1,5,6 |
| Pre-terminally differentiated effector memory RA+ 1 (pE1) | CD3+, CD8+, CD45RA+, CCR7-, CD27+, CD28+ | 1,5,6 |
| Pre-terminally differentiated effector memory RA+ 2 (pE2) | CD3+, CD8+, CD45RA+, CCR7-, CD27+, CD28- | 1,5,6 |
| Terminally differentiated effector memory RA+ effector (E) | CD3+, CD8+, CD45RA+, CCR7-, CD27-, CD28- | 1,5,6 |

References: **[1]** Koch, S.; Larbi, A.; Derhovanessian, E.; Ozcelik, D.; Naumova, E.; Pawelec, G. Multiparameter Flow Cytometric Analysis of CD4 and CD8 T Cell Subsets in Young and Old People. Immun Ageing 2008, 5, 6. <https://doi.org/10.1186/1742-4933-5-6>. **[2]** Larbi, A.; Fulop, T. From “Truly Naïve” to “Exhausted Senescent” T Cells: When Markers Predict Functionality. Cytometry Part A 2014, 85 (1), 25–35. <https://doi.org/10.1002/cyto.a.22351>; **[3]** Mahnke, Y. D.; Brodie, T. M.; Sallusto, F.; Roederer, M.; Lugli, E. The Who’s Who of T-Cell Differentiation: Human Memory T-Cell Subsets. Eur J Immunol 2013, 43 (11), 2797–2809. https://doi.org/10.1002/eji.201343751; **[4]** Okada, R.; Kondo, T.; Matsuki, F.; Takata, H.; Takiguchi, M. Phenotypic Classification of Human CD4+ T Cell Subsets and Their Differentiation. Int Immunol 2008, 20 (9), 1189–1199. <https://doi.org/10.1093/intimm/dxn075> ; **[5]** Romero, P., Zippelius, A., Kurth, I., Pittet, M. J., Touvrey, C., Iancu, E. M., Corthesy, P., Devevre, E., Speiser, D. E., & Rufer, N. (2007). Four functionally distinct populations of human effector-memory CD8+ T lymphocytes. Journal of immunology (Baltimore, Md.: 1950), 178(7), 4112–4119. <https://doi.org/10.4049/jimmunol.178.7.4112>; **[6]** Tilburgs, T.; Schonkeren, D.; Eikmans, M.; Nagtzaam, N. M.; Datema, G.; Swings, G. M.; Prins, F.; van Lith, J. M.; van der Mast, B. J.; Roelen, D. L.; Scherjon, S. A.; Claas, F. H. Human Decidual Tissue Contains Differentiated CD8+ Effector-Memory T Cells with Unique Properties. The Journal of Immunology 2010, 185 (7), 4470–4477. <https://doi.org/10.4049/jimmunol.0903597>

# **Supplementary Material 5. Gating strategy**

**A)**

**
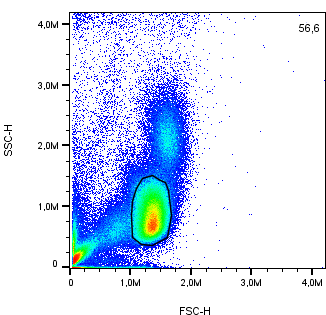

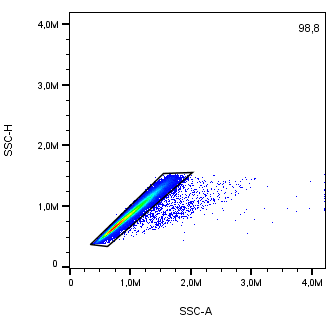

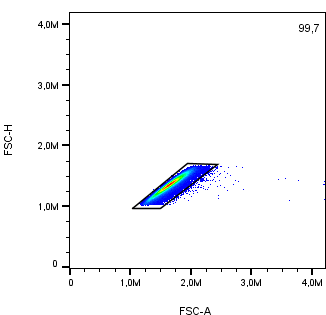

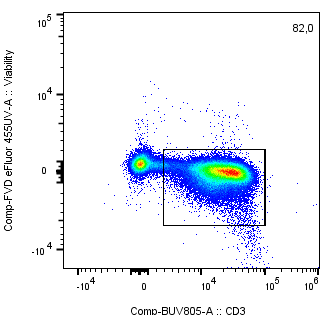

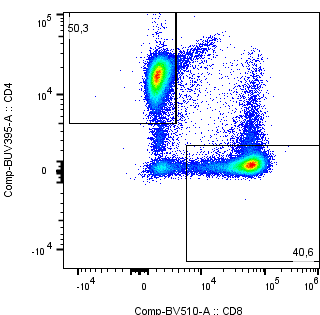
**

**CD8+**

**CD4+**

**B)**

**C)**

**Gated on N**

**Gated on CM**

**Gated on CD4+**

**
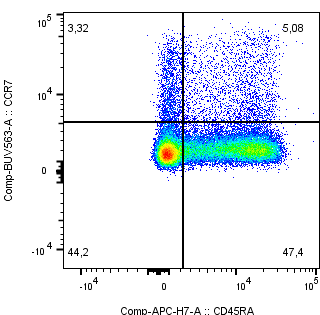

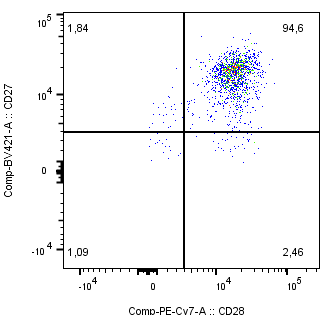

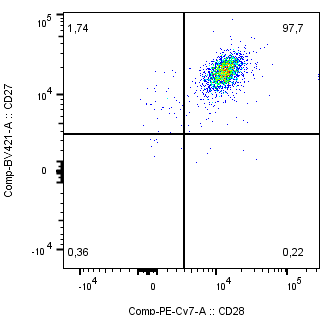
**

**CM**

**N**

**E**

**Th1**

**Th1-2**

**pE1**

**Th0-1**

**Gated on TEMRA**

**Gated on EM**

**EM**

**CM**

**N**

**TEMRA**

**
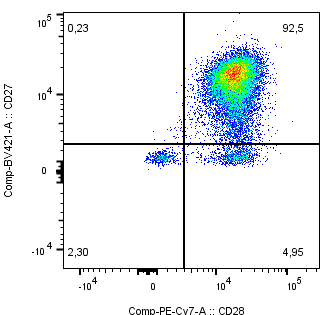

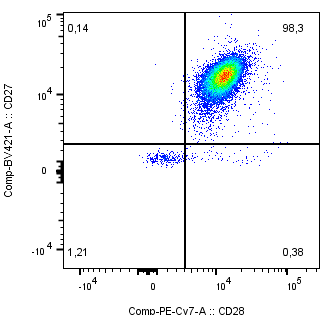
**

**D)**

**Gated on N**

**Gated on CM**

**E)**

**Gated on CD8+**

**
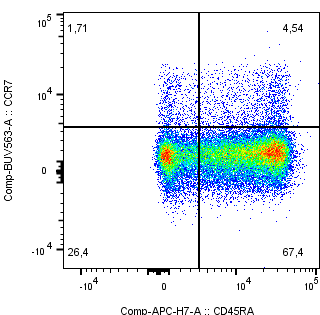

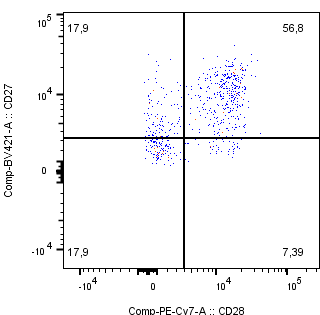

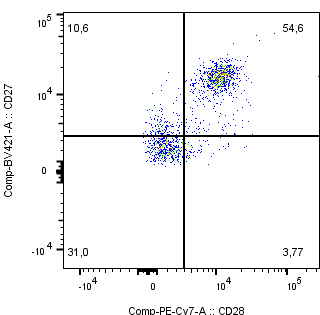
**

**CM**

**N**

**Gated on TEMRA**

**Gated on EM**

**N**

**TEMRA**

**EM**

**CM**

**
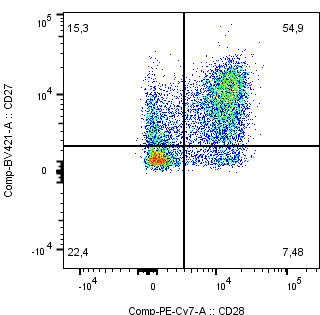

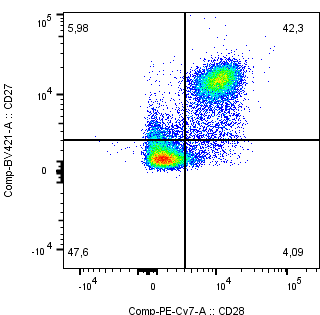
**

**EM2**

**EM3**

**EM4**

**EM1**

**pE2**

**E**

**pE1**

**Notes**: Differential expression of CD45RA, CCR7, CD27 and CD28 cell surface molecules on total CD4+ and CD8+ T cells from participants acquired on a Spectral Flow Cytometry Instrument (Cytek Biosciences). **A**, the FSC-Height (H) vs. Side scatter (SSC)-H is used to gate lymphocytes population. The FSC-H vs. FSC-A and SSC-H vs. SSC-A and are to gate on single cells and to exclude doublets. Next, the eFluor 455 UV-negative and the CD3-positive was used to gated live CD3+ lymphocytes and excluded dead or damage cells. **B**, CD4+ gated cells were separated into four subsets N (CCR7+CD45RA+), CM (CCR7+CD45RA-), EM (CCR7-CD45RA-), and TEMRA (CCR7-CD45RA+) based on CD45RA and CCR7 labeling. **C**, Each of these subsets was analyzed for CD27 and CD28 coexpression and seven subpopulations of CD4+ T cells could be distinguished: N (CD27+CD28+), CM (CD27+CD28+), EM Th0 and Th1 (CD27+CD28+), EM Th1 (CD27-CD28-) and EM Th1 and Th2 (CD27-CD28+), TEMRA pE1 (CD27+CD28+) and TEMRA E (CD27-CD28-). **D**, CD8+ gated cells were divided into four subsets N (CCR7+CD45RA+), CM (CCR7+CD45RA-), EM (CCR7-CD45RA-), and TEMRA (CCR7-CD45RA+) based on CD45RA and CCR7 labeling. **E,** Each of these subsets was analyzed for CD27 and CD28 coexpression and nine subpopulations of CD8+ T cells could be distinguished: N (CD27+CD28+), CM (CD27+CD28+), EM1 (CD27+CD28+), EM2 (CD27-CD28+), EM3 (CD27-CD28-), EM4 (CD27+CD28-), TEMRA pE1 (CD27+CD28+), TEMRA pE2 (CD27+CD28-) and TEMRA E (CD27-CD28-). On all these CD4+ T and CD8+ T subpopulations, the profile of intermediate activation (CD25 and HLADR), advanced activation (HLADR and CD38), intermediate senescence (CD57 and PD1) and advanced senescence (PD1 and TIM3) were analyzed. Abbreviations: N, naïve; CM, central memory; EM, effector memory; Th, T helper, TEMRA pE1, pre-terminally differentiated effector memory RA+ type 1; TEMRA pE2, pre-terminally differentiated effector memory RA+ type 2; TEMRA E, terminally differentiated effector memory RA+.

# **Supplementary Material 6. Markers of systemic inflammation evaluated in plasma of participants.**

| **Marker name** | **Official Protein Symbol** | **Official Gene Symbol** | **Official Full Name** |
| --- | --- | --- | --- |
| ***Th1/Th2*** |  |  |  |
| GM-CSF | GM-CSF | CSF2 | Granulocyte-macrophage colony-stimulating factor |
| IFN-γ | IFN- γ | IFNG | Interferon gamma |
| IL-1β | IL-1β | IL1B | Interleukin 1 beta |
| IL-2 | IL-2 | IL2 | Interleukin 2 |
| IL-4 | IL-4 | IL4 | Interleukin 4 |
| IL-5 | IL-5 | IL5 | Interleukin 5 |
| IL-6 | IL-6 | IL6 | Interleukin 6 |
| IL-8 | IL-8 | CXCL8 | C-X-C motif chemokine ligand 8 |
| IL-12p70 | IL-12p70 | IL12B | Interleukin 12 |
| IL-13 | IL-13 | IL13 | Interleukin 13 |
| IL-18 | IL-18 | IL18 | Interleukin 18 |
| TNF-α | TNF-α | TNF | Tumor necrosis factor alpha |
| ***Th9/Th17/Th22/Treg*** |  |  |  |
| IL-9 | IL-9 | IL9 | Interleukin 9 |
| IL-10 | IL-10 | IL10 | Interleukin 10 |
| IL-17A (CTLA-8) | IL-17A | IL-17A | Interleukin 17A |
| IL-21 | IL-21 | IL21 | Interleukin 21 |
| IL-22 | IL-22 | IL22 | Interleukin 22 |
| IL-23 | IL-23 | IL23A | Interleukin 23 |
| IL-27 | IL-27 | IL27 | Interleukin 27 |
| ***Inflammatory cytokines*** |  |  |  |
| IFN-α | IFN-α | IFNA1 | Interferon alpha 1 |
| IL-1α | IL-1α | IL1A | Interleukin 1 alpha |
| IL-1RA | IL-1RA | IL1RN | Interleukin-1 receptor antagonist |
| IL-7 | IL-7 | IL7 | Interleukin 7 |
| IL-15 | IL-15 | IL15 | Interleukin 15 |
| IL-31 | IL-31 | IL31 | Interleukin 31 |
| TNF beta | LTα | LTA | Lymphotoxin alpha |
| ***Chemokines*** |  |  |  |
| Eotaxin | Eotaxin | CCL11 | C-C motif chemokine ligand 11 |
| GRO-α | GRO-α | CXCL1 | C-X-C motif chemokine ligand 1 |
| IP-10 | CXCL10 | CXCL10 | C-X-C motif chemokine ligand 10 |
| MCP-1 | CCL2 | CCL2 | C-C motif chemokine ligand 2 |
| MIP-1α | CCL3 | CCL3 | C-C motif chemokine ligand 3 |
| MIP-1β | CCL4 | CCL4 | C-C motif chemokine ligand 4 |
| RANTES | CCL5 | CCL5 | C-C motif chemokine ligand 5 |
| SDF-1α | CXCL12 | CXCL12 | C-X-C motif chemokine ligand 12 |
| ***Growth factors*** |  |  |  |
| BDNF | BDNF | BDNF | Brain derived neurotrophic factor |
| EGF | EGF | EGF | Epidermal growth factor |
| FGF-2 | FGF-2 | FGF-2 | Fibroblast growth factor 2 |
| HGF | HGF | HGF | Hepatocyte growth factor |
| NGF-β | β-NGF | NGF | Nerve growth factor |
| LIF | LIF | LIF | Leukemia inhibitory factor |
| PDGF-BB | PDGF-BB | PDGFB | Platelet derived growth factor subunit B |
| PlGF-1 | PlGF-1 | PGF | Placental growth factor |
| SCF | KITLG | KITLG | KIT ligand |
| VEGF-A | VEGF-A | VEGFA | Vascular endothelial growth factor A |
| VEGF-D | VEGF-D | VEGFD | Vascular endothelial growth factor D |

**Supplementary Material 7. Epidemiological characteristics of all group of patients enrolled in this study.**

|  | **Total** | **LLV** | **SV** | **NHC** | **p^a^** | **p^b^** | **p^c^** | **p^d^** |
| --- | --- | --- | --- | --- | --- | --- | --- | --- |
| **No.** | 81 | 27 | 27 | 27 | -- | -- | -- | -- |
| **Sex at birth [n (%)]** |  |  |  |  |  |  |  |  |
| Male | 63 (77.80%) | 21 (77.80%) | 21 (77.80%) | 21 [77.80%] | 1.000 | 1.000 | 1.000 | 1.000 |
| **Ethnicity [n (%)]** |  |  |  |  |  |  |  |  |
| Caucasian | 76 (95.00%) | 25 (92.60%) | 25 (92.60%) | 27 (100.00%) | 1.000 | 1.000 | 1.000 | 1.000 |
| **Age (years)** | 53.00 [48.00-58.00] | 53.00 [47.50-58.00] | 54.00 [49.00-59.00] | 52.00 [49.00-55.00] | 0.502 | 0.775 | 0.376 | 0.275 |
| **Height (cm)** | 173.0 [166.0-178.0] | 170.0 [165.5-179.0] | 174.0 [165.8-178.0] | 173.0 [169.5-176.9] | 0.936 | 0.789 | 0.727 | 0.884 |
| **Weight (kg)** | 76.20 [69.43-89.72] | 76.00 [69.74-89.83] | 76.65 [68.29-89.86] | 76.20 [72.00-87.35] | 0.970 | 0.993 | 0.818 | 0.841 |
| **BMI (kg/m^2^)** | 25.86 [23.82-28.70] | 26.90 [24.65-28.70] | 24.90 [23.00-28.10] | 25.99 [24.00-29.00] | 0.373 | 0.228 | 0.713 | 0.237 |

Statistics: values are expressed as the absolute numbers (%) and median [interquartile range]. *P-values* were estimated by Kruskal-Wallis and Mann-Whitney U test for continuous variables and chi-squared or Fisher´s exact test for categorical variables. Statistically significant values are highlighted in bold. **a:** comparison between all the three groups enrolled in this study; **b**: comparison between LLV and SV; **c**: comparison between LLV and NHC; **d**: comparison between SV and NHC. Abbreviations: LLV, PWH with persistent low-level viremia (50-200 copies/mL); SV, PWH with suppressed viremia (<50 copies/mL); NHC, non-infected HIV controls; BMI, body mass index.

**Sup****plementary Material 8. Biochemical and metabolic characteristics of all group of patients enrolled in this study.**

|  | **Total** | **LLV** | **SV** | **NHC** | **p^a^** | **p^b^** | **p^c^** | **p^d^** |
| --- | --- | --- | --- | --- | --- | --- | --- | --- |
| **No.** | 81 | 27 | 27 | 27 | -- | -- | -- | -- |
| Platelets (cell/µL) | 229.00 [193.00- 278.750] | 250.00 [196.00-282.50] | 218.00 [203.50-298.00] | 228.00 [189.25-258.75] | 0.547 | 0.986 | 0.274 | 0.417 |
| **Lipid profile** |  |  |  |  |  |  |  |  |
| Glucose (mg/dL) | 93.50 [87.75- 101.25] | 94.00 [88.75- 116.00] | 97.50 [93.25- 102.00] | 88.50 [84.75- 92.00] | **<0.001** | 0.898 | **0.002** | **<0.001** |
| Glucose≥110 (%) | 12 (15.80%) | 8 (30.80%) | 4 (15.40%) | - | **0.009** | 0.324 | **0.004** | 0.111 |
| Creatinine (mg/dL) | 1.00 [0.88- 1.02] | 0.95 [0.90- 1.00] | 1.00 [0.90- 1.10] | 0.90 [0.70- 1.00] | 0.189 | 0.484 | 0.288 | 0.066 |
| TC (mg/dL) | 199.50 [168.25- 219.00] | 200.50 [165.00- 227.75] | 187.50 [157.25- 209.00] | 205.50 [182.75- 220.00] | 0.115 | 0.151 | 0.771 | **0.037** |
| TC≥200 (%) | 38 (50.00%) | 13 (50.00%) | 10 (38.50%) | 15 (62.50%) | 0.243 | 0.577 | 0.407 | 0.156 |
| LDL (mg/dL) | 117.80 [94.10- 136.45] | 115.00 [94.00- 147.00] | 114.00 [89.00- 131.00] | 118.50 [101.00- 136.75] | 0.719 | 0.547 | 0.904 | 0.435 |
| LDL≥130 (%) | 28 (37.80%) | 10 (40.00%) | 9 (36.00%) | 9 (37.50%) | 1.000 | 1.000 | 1.000 | 1.000 |
| HDL (mg/dL) | 49.00 [41.00- 60.00] | 47.00 [41.00- 57.00] | 43.00 [35.50- 50.25] | 60.00 [49.75- 71.50] | **<0.001** | 0.122 | **0.006** | **<0.001** |
| HDL>40 (%) | 16 (21.30%) | 5 (20.00%) | 10 (38.5%) | 1 (4.2%) | **0.009** | 0.220 | 0.189 | **0.005** |
| TG (mg/dL) | 121.00 [87.50- 159.75] | 128.00 [95.75- 178.00] | 127.00 [100.25- 167.50] | 97.00 [71.50- 138.75] | **0.020** | 0.861 | **0.016** | **0.016** |
| TG≥200 (%) | 11 (14.90%) | 5 (20.80%) | 6 (23.10%) | - | **0.029** | 1.000 | 0.050 | **0.023** |
| LDL/HDL (mg/dL) | 2.38 [1.87- 2.73] | 2.39 [1.85- 2.73] | 2.44 [2.00- 3.14] | 2.19 [1.45- 2.59] | 0.113 | 0.547 | 0.174 | **0.038** |
| AI (%) | 3.90 [3.32- 4.45] | 4.00 [3.57- 4.56] | 3.97 [3.66- 4.83] | 3.64 [2.72- 4.11] | **0.022** | 0.692 | 0.050 | **0.006** |
| AIP (%) | 0.43 [0.28- 0.59] | 0.48 [0.22- 0.64] | 0.48 [0.37- 0.64] | 0.31 [0.22- 0.45] | 0.124 | 0.560 | 0.169 | **0.039** |
| **Biochemical parameters of liver function** | | | | | | | | |
| AST (mg/dL) | 20.00 [17.00- 26.00] | 23.50 [19.00- 29.00] | 21.00 [16.50- 26.00] | 19.00 [15.75- 20.25] | **0.025** | 0.199 | **0.005** | 0.179 |
| AST≥40 (%) | 3 (3.90%) | 3 (11.50%) | -- | - | 0.063 | 0.111 | 0.236 | -- |
| ALT (mg/dL) | 24.00 [18.00- 34.00] | 32.00 [22.75- 45.75] | 30.00 [20.00- 41.00] | 17.00 [13.75- 21.00] | **<0.001** | 0.373 | **<0.001** | **<0.001** |
| ALT≥40 (%) | 14 (18.40%) | 8 (30.8%) | 6 (23.1%) | - | **0.008** | 0.755 | **0.004** | **0.023** |
| GGT (mg/dL) | 25.00 [19.75- 34.50] | 25.50 [22.25- 39.50] | 25.50 [22.00- 35.50] | 21.50 [16.00- 28.50] | 0.124 | 0.564 | 0.058 | 0.122 |
| GGT≥50 (%) | 7 (9.20%) | 6 (23.10%) | 1 (3.80%) | - | **0.014** | 0.099 | **0.023** | 1.000 |
| TB (mg/dL) | 0.60 [0.40- 0.80] | 0.60 [0.50- 0.80] | 0.60 [0.43- 1.00] | 0.54 [0.37- 0.70] | 0.235 | 0.512 | 0.188 | 0.134 |
| Hemoglobin (g/dL) | 150.35 [140.83-160.10] | 150.30 [140.45-160.00] | 150.60 [150.10-160.40] | 150.10 [140.45-160.02] | 0.147 | 0.113 | 0.727 | 0.080 |

Statistics: values are expressed as the absolute numbers (%) and median [interquartile range]. *P-values* were estimated by Kruskal-Wallis and Mann-Whitney U test for continuous variables and chi-squared or Fisher´s exact test for categorical variables0. Statistically significant values are highlighted in bold. **a:** comparison between all the three groups enrolled in this study; **b**: comparison between LLV and SV; **c**: comparison between LLV and NHC; **d**: comparison between SV and NHC. Abbreviations: LLV, PWH with persistent low-level viremia (50-200 copies/mL); SV: PWH with virologic suppression; NHC, non-infected HIV controls; TC, total cholesterol; LDL, low-density lipoprotein; HDL, high-density lipoprotein; TG, triglycerides; AI, Atherogenic index (low risk <5% for men and <40.5% for women, moderate risk 5-9 men and 40.5-7 women); AIP, Atherogenic index for plasma (high risk AIP>0.21); AST, Aspartato aminotransferasa; ALT, Alanina aminotransferase; GGT, Gamma glutamil transferasa; TB, total bilirubin.

# **Supplementary Material 9. Comparison of T-cell development, activation, and senescence levels of CD4+ T-cells between all groups of study.**

| **CD4+ subpopulations** | **LLV** | **SV** | **NHC** | **aAMR^a^**  **[IC95%]** | **p^a^** | **q^a^** | **aAMR^b^**  **[IC95%]** | **p^b^** | **q^b^** | **aAMR^c^**  **[IC95%]** | **p^c^** | **q^c^** |  |
| --- | --- | --- | --- | --- | --- | --- | --- | --- | --- | --- | --- | --- | --- |
| **Development** |  |  |  |  |  |  |  |  |  |  |  |  |  |
| CD4+ | 47.70 [41.05-55.55] | 53.75 [44.55-59.70] | 60.10 [53.50-65.50] | 0.67 [0.51-0.87] | **0.005** | **0.051** | 0.71 [0.55-0.91] | **0.010** | **0.092** | 0.79 [0.59-1.06] | 0.118 | 0.915 |  |
| TEMRA | 36.10 [22.20-44.75] | 33.30 [29.27-45.45] | 42.00 [26.95-49.65] | 0.76 [0.56-1.04] | 0.092 | 0.385 | 0.65 [0.46-0.90] | **0.013** | **0.094** | 1.17 [0.82-1.66] | 0.391 | 0.808 |  |
| **Activation (HLADR+)** |  |  |  |  |  |  |  |  |  |  |  |  |  |
| N | 9.39 [5.70-19.85] | 8.84 [7.08-12.27] | 4.05 [2.97-8.97] | 1.78 [1.14-2.78] | **0.014** | **0.113** | 1.60 [1.07-2.41] | **0.029** | 0.297 | 1.16 [0.78-1.71] | 0.463 | 0.956 |  |
| EM Th1 | 45.30 [31.50-75.45] | 44.15 [24.25-51.00] | 26.55 [14.90-41.25] | 2.54 [1.47-4.39] | **0.002** | **0.028** | 1.62 [1.03-2.55] | **0.044** | 0.317 | 1.75 [1.05-2.93] | **0.037** | 0.590 |  |
| TEMRA E | 62.50 [30.40-74.30] | 36.60 [27.90-56.05] | 21.20 [8.40-32.00] | 3.30 [1.89-5.78] | **<0.001** | **0.005** | 2.29 [1.41-3.74] | **0.002** | 0.157 | 1.65 [0.97-2.80] | 0.072 | 0.651 |  |
| **Activation (CD38+)** |  |  |  |  |  |  |  |  |  |  |  |  |  |
| CD4+ | 25.40 [19.70-29.60] | 16.20 [11.73-24.90] | 12.40 [7.69-23.85] | 1.68 [1.20-2.35] | **0.004** | **0.055** | 1.11 [0.81-1.53] | 0.524 | 0.727 | 1.54 [1.17-2.03] | **0.004** | **0.100** |  |
| N | 48.90 [38.65-59.85] | 18.05 [14.10-26.15] | 18.20 [11.50-31.10] | 2.77 [1.90-4.90] | **<0.001** | **<0.001** | 0.81 [0.54-1.21] | 0.299 | 0.570 | 3.75 [2.83-4.98] | **<0.001** | **<0.001** |  |
| CM | 18.70 [12.40-21.45] | 13.40 [10.90-18.82] | 8.62 [7.36-16.25] | 1.69 [1.26-2.27] | **0.001** | **0.024** | 1.39 [1.04-1.87] | **0.034** | 0.297 | 1.22 [0.98-1.53] | 0.085 | 0.651 |  |
| EM Th0-1 | 11.70 [8.30-14.25] | 10.45 [7.33-13.62] | 6.96 [5.34-9.23] | 1.47 [1.10-1.95] | **0.012** | **0.113** | 1.32 [1.03-1.70] | **0.035** | 0.297 | 1.07 [0.81-1.40] | 0.646 | 0.956 |  |
| TEMRA pE1 | 46.70 [40.20-57.30] | 12.05 [7.59-18.20] | 12.55 [9.72-25.15] | 3.55 [2.35-5.38] | **<0.001** | **<0.001** | 0.66 [0.43-1.01] | 0.066 | 0.344 | 6.16 [4.43-8.57] | **<0.001** | **<0.001** |  |
| **Late activation (HLADR+CD8+)** |  |  |  |  |  |  |  |  |  |  |  |  |  |
| N | 3.45 [2.05-5.07] | 3.27 [2.18-5.94] | 1.54 [0.71-6.03] | 1.61 [1.14-2.28] | **0.010** | **0.110** | 1.41 [1.00-2.01] | 0.059 | 0.335 | 1.17 [0.87-1.59] | 0.307 | 0.956 |  |
| EM Th1 | 4.60 [2.74-9.48] | 3.97 [2.17-6.10] | 2.07 [0.74-3.35] | 1.75 [1.08-2.83] | **0.027** | 0.192 | 1.65 [1.18-2.31] | **0.006** | 0.225 | 1.21 [0.79-1.86] | 0.391 | 0.956 |  |
| **Senescence (PD1+)** |  |  |  |  |  |  |  |  |  |  |  |  |  |
| CD4+ | | 15.40 [10.75-20.10] | 10.02 [7.94-13.65] | 9.45 [7.06-11.05] | 1.61 [1.24-2.08] | **0.001** | **0.002** | 1.14 [0.92-1.43] | 0.241 | 0.344 | 1.35 [1.05-1.73] | **0.025** | **0.144** |
| N | 74.90 [68.55-82.70] | 78.55 [72.10-85.08] | 39.60 [19.60-61.70] | 2.71 [1.69-4.32] | **<0.001** | **0.001** | 3.49 [2.23-5.46] | **<0.001** | **<0.001** | 0.78 [0.56-1.09] | 0.158 | 0.382 |  |
| CM | | 59.50 [50.35-69.45] | 55.15 [41.52-63.25] | 67.60 [40.30-77.30] | 1.88 [1.24-2.85] | **0.005** | **0.011** | 2.57 [1.73-3.83] | **<0.001** | **<0.001** | 0.72 [0.53-0.97] | **0.036** | 0.159 |
| EM Th0-1 | 11.00 [6.96-16.45] | 15.15 [10.88-21.27] | 7.18 [5.87-10.73] | 1.18 [0.78-1.77] | 0.440 | 0.512 | 1.75 [1.20-2.56] | **0.006** | **0.017** | 0.57 [0.42-0.76] | **<0.001** | **0.011** |  |
| TEMRA pE1 | | 1.01 [0.42-1.60] | 1.93 [1.08-3.84] | 0.56 [0.32-1.26] | 1.26 [0.81-1.96] | 0.321 | **0.**410 | 2.61 [1.67-4.07] | **<0.001** | **0.001** | 0.47 [0.31-0.72] | **0.001** | **0.019** |
| **Senescence (HAVCR2+)** | |  |  |  |  |  |  |  |  |  |  |  |  |
| CD4+ | | 3.93 [2.82-5.88] | 3.26 [2.19-4.65] | 2.54 [1.77-3.74] | 1.52 [1.19-1.93] | **0.001** | **0.004** | 1.18 [0.94-1.48] | 0.168 | 0.270 | 1.29 [1.05-1.58] | **0.019** | **0.125** |
| N | | 36.70 [27.10-43.55] | 30.25 [21.98-37.60] | 21.40 [13.20-29.40] | 1.73 [1.15-2.61] | **0.012** | **0.025** | 1.29 [0.81-2.03] | 0.288 | 0.397 | 1.40 [0.95-2.06] | 0.093 | 0.125 |
| EM Th0-1 | | 0.91 [0.66-1.71] | 0.55 [0.38-0.91] | 0.38 [0.27-0.72] | 1.97 [1.41-2.77] | **<0.001** | **0.001** | 1.23 [0.84-1.80] | 0.304 | 0.404 | 1.24 [0.75-1.50] | 0.051 | 0.159 |
| EM Th1 | | 2.30 [1.50-3.51] | 2.77 [1.60-5.59] | 0.61 [0.27-1.26] | 2.53 [1.63-3.93] | **<0.001** | **0.001** | 2.59 [1.56-4.29] | **0.001** | **0.003** | 0.80 [0.53-1.22] | 0.303 | 0.597 |
| EM Th1-2 | 1.56 [1.17-2.62] | 0.86 [0.55-1.44] | 0.71 [0.30-1.07] | 1.76 [1.27-2.46] | **0.002** | **0.004** | 1.25 [0.82-1.90] | 0.297 | 0.403 | 1.37 [0.95-1.98] | 0.097 | 0.278 |  |
| TEMRA pE1 | 1.20 [0.72-1.84] | 0.70 [0.35-1.18] | 0.38 [0.26-0.54] | 2.53 [1.69-3.78] | **<0.001** | **<0.001** | 1.62 [1.16-2.27] | **0.007** | **0.020** | 1.73 [1.21-2.49] | **0.005** | **0.043** |  |
| TEMRA E | 3.57 [2.64-10.80] | 1.78 [1.33-9.70] | 0.63 [0.00-1.34] | 2.56 [1.35-4.88] | **0.007** | **0.015** | 1.78 [0.92-3.43] | 0.095 | 0.178 | 1.27 [0.72-2.25] | 0.419 | 0.657 |  |
| **Intermediate senescence (PD1+CD57+)** |  |  |  |  |  |  |  |  |  |  |  |  |  |
| N | 3.19 [1.50-8.82] | 3.59 [1.98-5.87] | 2.39 [0.89-4.06] | 1.50 [0.96-2.32] | 0.080 | 0.132 | 1.54 [1.07-2.21] | **0.026** | **0.063** | 1.03 [0.69-1.55] | 0.886 | 0.998 |  |
| CM | 18.50 [10.66-27.55] | 16.40 [9.37-26.60] | 13.50 [7.56-25.80] | 1.52 [1.07-2.16] | **0.023** | **0.046** | 1.39 [1.04-1.85] | **0.029** | **0.069** | 1.13 [0.83-1.53] | 0.446 | 0.674 |  |
| EM Th1-2 | 3.51 [1.44-6.15] | 3.23 [2.38-4.28] | 1.60 [0.91-2.99] | 1.23 [0.82-1.83] | 0.322 | 0.410 | 1.50 [1.02-2.20] | **0.044** | **0.102** | 0.85 [0.60-1.21] | 0.376 | 0.639 |  |
| **Advanced senescence (PD1+HAVCR2+)** |  |  |  |  |  |  |  |  |  |  |  |  |  |
| CD4+ | 4.81 [3.41-6.42] | 3.22 [2.23-4.94] | 2.73 [1.77-3.98] | 1.52 [1.18-1.97] | **0.002** | **0.006** | 1.12 [0.86-1.46] | 0.398 | 0.489 | 1.41 [1.12-1.76] | **0.005** | **0.043** |  |
| N | 67.10 [45.55-80.65] | 62.60 [48.25-74.00] | 28.85 [9.18-48.75] | 4.08 [2.48-6.70] | **<0.001** | **<0.001** | 3.55 [2.25-5.60] | **<0.001** | **<0.001** | 1.23 [0.87-1.73] | 0.253 | 0.533 |  |
| CM | 79.80 [71.50-84.55] | 72.55 [69.75-79.28] | 37.70 [27.10-50.00] | 4.15 [2.67-6.47] | **<0.001** | **<0.001** | 3.24 [2.19-4.80] | **<0.001** | **<0.001** | 1.39 [1.00-1.92] | 0.053 | 0.184 |  |
| EM Th0-1 | 1.10 [0.60-2.18] | 0.44 [0.25-0.86] | 0.06 [0.02-0.13] | 4.04 [2.46-6.64] | **<0.001** | **<0.001** | 2.38 [1.44-3.93] | **0.001** | **0.004** | 1.77 [1.24-2.08] | **0.007** | **0.054** |  |
| TEMRA E | 0.92 [0.00-2.30] | 0.52 [0.00-1.33] | 0.00 [0.00-0.00] | 0.98 [0.44-2.15] | 0.954 | 0.954 | 0.53 [0.25-1.13] | 0.111 | 0.189 | 1.78 [1.11-2.83] | **0.021** | **0.129** |  |

**Note:** Values are expressed as median of cell frequency [interquartile range]. Statistics: The AMR values [interquartile range] were obtained using a GALMSS with zero-one inflated beta distribution and multivariate analysis was performed with age, sex, and antiretroviral therapy. **a**: comparison between LLV and NHC groups; **b**: comparison between SV and NHC groups; **c**: comparison between LLV and SV groups. Abbreviations: LLV, PWH with persistent low-level viremia (50-200 copies/mL); SV: PWH with virologic suppression (<50 copies/mL); NHC; non-infected HIV controls; aAMR, adjusted Arithmetic Median Ratio; q, corrected level of significance by false discovery rate; N, naïve T-cells; CM, central memory T cells; EM, memory effector T-cells, TEMRA pE1, pre-terminally differentiated effector memory RA+ 1 T cells; TEMRA pE2, intermediate differentiated effector memory RA+ 2 T cells; TEMRA E, terminally differentiated effector memory RA+ T cells; HAVCR2 also known as TIM3.

# **Supplementary Material 10. Comparison of T-cell development, activation, and senescence levels of CD8+ T-cells between all groups of study.**

| **CD8+ subpopulations** | **LLV** | **SV** | **NHC** | **aAMR^a^**  **[IC95%]** | **p^a^** | **q^a^** | **aAMR^b^**  **[IC95%]** | **p^b^** | **q^b^** | **aAMR^c^**  **[IC95%]** | **p^c^** | **q^c^** |  |
| --- | --- | --- | --- | --- | --- | --- | --- | --- | --- | --- | --- | --- | --- |
| **Development** |  |  |  |  |  |  |  |  |  |  |  |  |  |
| CD8+ | 45.60 [37.40-53.45] | 38.35 [35.27-48.73] | 31.60 [26.40-35.20] | 1.60 [1.21-2.12] | **0.002** | **0.013** | 1.49 [1.14-1.94] | **0.005** | **0.079** | 1.25 [0.95-1.66] | 0.120 | 0.374 |  |
| N | 1.67 [1.15-2.76] | 2.54 [1.64-4.03] | 2.89 [1.67-3.67] | 0.67 [0.48-0.92] | **0.019** | **0.077** | 0.96 [0.71-1.28] | 0.761 | 0.888 | 0.67 [0.50-0.89] | **0.034** | 0.285 |  |
| EM | 55.00 [40.75-69.30] | 48.65 [44.10-54.35] | 39.30 [32.85-45.90] | 1.84 [1.33-2.53] | **0.001** | **0.008** | 1.41 [1.09-1.82] | **0.011** | **0.079** | 1.32 [1.00-1.74] | 0.054 | 0.285 |  |
| EM1 | 45.60 [37.25-56.05] | 55.80 [39.17-59.98] | 45.60 [32.80-67.65] | 1.06 [0.68-1.67] | 0.789 | 0.830 | 1.31 [0.85-2.03] | 0.226 | 0.475 | 0.66 [0.46-0.96] | **0.034** | 0.285 |  |
| TEMRA | 35.60 [26.10-51.90] | 46.15 [38.98-50.00] | 55.00 [46.25-59.55] | 0.57 [0.42-0.77] | **0.001** | **0.008** | 0.72 [0.57-0.91] | **0.010** | **0.079** | 0.78 [0.59-1.02] | 0.075 | 0.313 |  |
| CD4/CD8 ratio | 1.04 [0.77-1.49] | 1.43 [0.92-1.69] | 1.91 [1.59-2.44] | 0.64 [0.47-0.87] | **0.007** | **0.051** | 0.68 [0.51-0.91] | **0.013** | **0.092** | 0.79 [0.59-1.05] | 0.113 | 0.915 |  |
| **Activation (HLADR+)** |  |  |  |  |  |  |  |  |  |  |  |  |  |
| CD8+ | 45.90 [32.20-61.00] | 42.75 [37.70-50.05] | 22.00 [14.55-32.15] | 2.22 [1.42-3.49] | **0.001** | **0.015** | 2.06 [1.45-2.94] | **<0.001** | **0.005** | 1.40 [0.91-2.15] | 0.131 | 0.923 |  |
| CM | 40.60 [27.15-57.75] | 32.25 [21.88-40.27] | 21.00 [8.82-32.60] | 2.10 [1.34-3.29] | **0.002** | **0.028** | 1.54 [1.01-2.32] | **0.049** | 0.181 | 1.47 [1.01-2.13] | **0.048** | 0.993 |  |
| EM2 | 65.00 [45.90-77.75] | 52.10 [44.92-61.00] | 25.00 [13.70-48.50] | 2.94 [1.70-5.06] | **<0.001** | **0.015** | 2.18 [1.37-3.46] | **0.002** | **0.020** | 1.50 [0.99-2.29] | 0.064 | 0.923 |  |
| EM3 | 59.60 [30.75-74.55] | 47.90 [30.40-56.68] | 31.20 [6.87-51.80] | 2.35 [1.28-4.33] | **0.009** | **0.084** | 1.68 [0.98-2.89] | 0.066 | 0.202 | 1.59 [0.99-2.54] | 0.061 | 0.923 |  |
| EM4 | 42.30 [32.65-64.95] | 49.40 [38.12-55.30] | 25.10 [12.82-38.35] | 1.86 [1.10-3.15] | **0.025** | **0.120** | 1.68 [1.06-2.67] | **0.034** | 0.163 | 1.17 [0.78-1.75] | 0.450 | 0.980 |  |
| TEMRA pE2 | 40.50 [21.55-62.05] | 37.10 [20.25-45.82] | 22.00 [10.40-39.50] | 1.83 [1.05-3.19] | **0.039** | **0.143** | 1.32 [0.80-2.20] | 0.284 | 0.474 | 1.48 [0.93-2.35] | 0.107 | 0.923 |  |
| TEMRA E | 60.90 [38.60-77.40] | 45.40 [29.23-55.85] | 36.70 [22.90-55.55] | 1.99 [1.19-3.34] | **0.012** | **0.089** | 1.12 [0.73-1.71] | 0.616 | 0.800 | 1.85 [1.15-2.97] | **0.015** | 0.736 |  |
| **Activation (CD38+)** | |  |  |  |  |  |  |  |  |  |  |  |  |
| N | | 14.00 [11.45-24.20] | 14.00 [8.93-23.62] | 11.65 [4.63-17.52] | 1.72 [1.12-2.63] | **0.017** | **0.098** | 1.67 [1.07-2.61] | **0.030** | 0.163 | 1.01 [0.71-1.44] | 0.965 | 0.993 |
| EM2 | 5.31 [2.63-7.18] | 5.70 [3.43-8.97] | 0.50 [0.08-2.27] | 2.65 [1.54-4.56] | **0.001** | **0.015** | 3.39 [2.04-5.63] | **<0.001** | **0.002** | 0.95 [0.61-1.49] | 0.823 | 0.980 |  |
| EM3 | 4.26 [2.02-9.01] | 5.58 [2.36-8.80] | 1.61 [0.17-5.33] | 2.11 [1.23-3.59] | **0.009** | **0.084** | 2.99 [1.85-4.84] | **<0.001** | **0.002** | 0.65 [0.40-1.05] | 0.083 | 0.993 |  |
| EM4 | 2.13 [1.62-3.08] | 2.43 [1.52-4.20] | 0.72 [0.39-2.79] | 1.63 [1.06-2.52] | **0.032** | **0.136** | 1.59 [1.06-2.39] | **0.029** | 0.163 | 1.06 [0.73-1.53] | 0.760 | 0.980 |  |
| TEMRA pE2 | 6.15 [4.18-10.16] | 5.57 [3.04-11.77] | 1.65 [0.58-3.59] | 2.51 [1.55-4.05] | **0.001** | **0.015** | 2.36 [1.51-3.70] | **<0.001** | **0.006** | 1.16 [0.77-1.73] | 0.482 | 0.980 |  |
| **Late activation (HLADR+CD38+)** |  |  |  |  |  |  |  |  |  |  |  |  |  |
| EM1 | 3.09 [2.14-5.15] | 3.23 [1.95-5.37] | 1.79 [1.09-3.08] | 1.55 [1.01-2.39] | 0.052 | 0.165 | 1.59 [1.06-2.39] | **0.031** | **0.129** | 0.99 [0.69-1.42] | 0.957 | 0.994 |  |
| EM2 | 6.01 [2.84-8.53] | 6.80 [3.64-9.67] | 2.94 [1.19-4.53] | 1.69 [1.02-2.81] | 0.050 | 0.165 | 2.02 [1.30-3.13] | **0.003** | **0.028** | 0.96 [0.61-1.51] | 0.856 | 0.980 |  |
| EM3 | 6.95 [2.72-13.75] | 6.96 [3.38-10.75] | 4.77 [1.21-6.90] | 1.39 [0.84-2.32] | 0.212 | 0.388 | 1.61 [1.06-2.45] | **0.032** | **0.129** | 0.97 [0.62-1.49] | 0.874 | 0.993 |  |
| **Senescence (PD1+)** |  |  |  |  |  |  |  |  |  |  |  |  |  |
| CD8+ | 12.90 [9.57-17.60] | 14.35 [10.28-17.33] | 5.35 [3.70-7.88] | 1.96 [1.27-3.04] | **0.004** | **0.016** | 1.77 [1.16-2.70] | **0.011** | **0.044** | 1.13 [0.88-1.45] | 0.346 | 0.617 |  |
| N | 61.10 [37.50-69.20] | 50.40 [39.03-62.38] | 35.75 [13.30-55.35] | 1.97 [1.21-3.19] | **0.009** | **0.029** | 1.62 [1.02-2.55] | **0.045** | **0.136** | 1.26 [0.91-1.73] | 0.165 | 0.434 |  |
| EM1 | 8.78 [6.29-16.85] | 12.25 [7.70-19.62] | 8.99 [5.65-13.70] | 0.99 [0.71-1.39] | 0.965 | 0.975 | 1.34 [0.95-1.88] | 0.098 | 0.251 | 0.64 [0.45-0.91] | **0.017** | **0.088** |  |
| EM2 | 11.10 [6.24-17.75] | 21.30 [10.97-25.90] | 8.81 [4.91-20.90] | 0.85 [0.50-1.45] | 0.555 | 0.712 | 1.19 [0.72-1.95] | 0.503 | 0.671 | 0.59 [0.39-0.87] | **0.012** | **0.069** |  |
| EM4 | 3.66 [1.52-6.97] | 10.80 [6.80-15.63] | 5.96 [3.09-10.68] | 0.59 [0.36-0.95] | **0.037** | **0.082** | 1.28 [0.83-1.97] | 0.280 | 0.444 | 0.41 [0.28-0.62] | **<0.001** | **0.002** |  |
| TEMRA pE1 | 1.13 [0.55-2.04] | 2.25 [1.33-4.00] | 1.60 [0.84-3.00] | 0.61 [0.40-0.92] | **0.019** | **0.055** | 1.04 [0.70-1.55] | 0.858 | 0.943 | 0.43 [0.29-0.64] | **<0.001** | **0.002** |  |
| TEMRA pE2 | 3.04 [1.36-6.16] | 2.88 [1.47-5.44] | 4.06 [2.50-6.40] | 0.63 [0.42-0.95] | **0.032** | **0.074** | 0.60 [0.42-0.87] | **0.011** | **0.044** | 1.00 [0.65-1.55] | 0.999 | 0.999 |  |
| **Senescence (HAVCR2+)** |  |  |  |  |  |  |  |  |  |  |  |  |  |
| CD8+ | | 3.07 [2.40-4.42] | 2.24 [1.41-2.81] | 1.77 [1.15-2.34] | 1.71 [1.31-2.22] | **<0.001** | **0.002** | 1.17 [0.87-1.58] | 0.301 | 0.457 | 1.46 [1.14-1.85] | **0.004** | **0.027** |
| N | | 27.40 [17.60-40.75] | 25.55 [15.48-36.25] | 6.96 [3.28-12.53] | 3.82 [2.59-5.63] | **<0.001** | **<0.001** | 3.22 [2.11-4.92] | **<0.001** | **<0.001** | 1.15 [0.83-1.59] | 0.418 | 0.663 |
| CM | | 25.10 [13.15-30.95] | 27.30 [14.80-38.27] | 15.50 [9.41-24.90] | 1.53 [1.07-2.20] | **0.026** | **0.064** | 1.93 [1.15-3.25] | **0.017** | **0.059** | 0.60 [0.36-0.99] | 0.054 | 0.226 |
| EM1 | | 2.92 [1.53-4.09] | 1.00 [0.80-1.66] | 0.82 [0.48-1.24] | 2.73 [1.85-4.04] | **<0.001** | **<0.001** | 1.28 [0.93-1.75] | 0.134 | 0.285 | 2.34 [1.66-3.31] | **<0.001** | **<0.001** |
| EM2 | | 3.26 [2.29-4.30] | 1.77 [1.43-2.76] | 1.35 [0.63-3.04] | 1.62 [1.16-2.26] | **0.008** | **0.025** | 0.95 [0.67-1.35] | 0.784 | 0.900 | 1.74 [1.29-2.36] | **0.001** | **0.008** |
| EM3 | | 0.92 [0.70-1.54] | 0.92 [0.68-1.35] | 0.63 [0.28-0.86] | 1.56 [1.08-2.24] | **0.022** | **0.058** | 1.46 [0.96-2.22] | 0.082 | 0.222 | 1.05 [0.74-1.50] | 0.785 | 0.855 |
| EM4 | | 2.63 [2.10-3.50] | 1.27 [0.70-1.98] | 0.60 [0.20-1.33] | 3.17 [2.11-4.76] | **<0.001** | **<0.001** | 1.48 [0.93-2.36] | 0.106 | 0.264 | 1.99 [1.38-2.87] | **0.001** | **0.007** |
| TEMRA pE1 | | 2.07 [1.50-3.65] | 1.30 [1.04-1.95] | 0.63 [0.38-1.23] | 2.16 [1.46-3.19] | **<0.001** | **0.002** | 1.47 [0.87-2.47] | 0.155 | 0.308 | 1.16 [0.72-1.86] | 0.556 | 0.742 |
| TEMRA pE2 | 3.99 [2.58-4.67] | 1.62 [1.22-2.49] | 1.45 [0.69-2.09] | 2.28 [1.59-3.26] | **<0.001** | **0.001** | 1.10 [0.77-1.57] | 0.614 | 0.758 | 2.22 [1.64-3.00] | **<0.001** | **<0.001** |  |
| **Intermediate senescence (PD1+CD57+)** |  |  |  |  |  |  |  |  |  |  |  |  |  |
| EM1 | 6.58 [3.99-12.45] | 7.31 [4.32-10.35] | 4.44 [2.88-6.25] | 1.64 [1.12-2.40] | **0.015** | **0.044** | 1.43 [1.02-2.00] | **0.046** | **0.136** | 1.17 [0.84-1.64] | 0.361 | 0.617 |  |
| EM3 | 5.19 [4.13-10.46] | 4.46 [3.40-7.45] | 10.50 [5.42-20.00] | 0.80 [0.50-1.30] | 0.373 | 0.532 | 0.54 [0.34-0.85] | **0.011** | **0.044** | 1.35 [0.84-2.16] | 0.221 | 0.513 |  |
| TEMRA E | 7.23 [2.32-22.50] | 4.69 [2.44-11.80] | 11.38 [5.00-19.50] | 0.79 [0.54-1.15] | 0.222 | 0.358 | 0.61 [0.41-0.90] | **0.016** | **0.057** | 1.36 [1.01-1.85] | 0.052 | 0.226 |  |
| **Advance senescence (PD1+HAVCR2+)** |  |  |  |  |  |  |  |  |  |  |  |  |  |
| CD8+ | 3.45 [2.29-4.42] | 3.66 [2.28-4.48] | 1.18 [0.43-2.23] | 1.85 [1.23-2.80] | **0.005** | **0.020** | 1.81 [1.20-2.72] | **0.007** | **0.030** | 0.91 [0.73-1.15] | 0.443 | 0.682 |  |
| N | 67.10 [45.55-80.65] | 62.60 [48.25-74.00] | 28.85 [9.18-48.75] | 3.20 [1.82-5.61] | **<0.001** | **0.002** | 2.69 [1.58-4.59] | **0.001** | **0.006** | 1.27 [0.87-1.85] | 0.220 | 0.513 |  |
| CM | 79.80 [71.50-84.55] | 72.55 [69.75-79.28] | 37.70 [27.10-50.00] | 2.33 [1.38-3.94] | **0.003** | **0.012** | 1.93 [1.17-3.18] | **0.013** | **0.050** | 1.26 [0.84-1.88] | 0.264 | 0.550 |  |
| EM3 | 0.55 [0.37-1.31] | 0.26 [0.11-0.59] | 0.00 [0.00-0.04] | 3.25 [1.72-6.16] | **0.001** | **0.004** | 1.69 [0.89-3.21] | 0.118 | 0.270 | 1.99 [1.33-2.99] | **0.002** | **0.014** |  |
| TEMRA pE1 | 0.81 [0.46-1.43] | 0.69 [0.37-1.05] | 0.07 [0.01-0.17] | 2.75 [2.41-6.05] | **<0.001** | **0.001** | 2.22 [1.34-3.67] | **0.003** | **0.016** | 1.22 [0.83-1.81] | 0.318 | 0.589 |  |
| TEMRA E | 0.45 [0.28-0.92] | 0.09 [0.02-0.18] | 0.00 [0.00-0.01] | 4.12 [2.02-8.42] | **<0.001** | **0.002** | 1.70 [0.88-3.32] | 0.124 | 0.276 | 2.61 [1.67-4.07] | **<0.001** | **0.002** |  |

**Note:** Values are expressed as median of cell frequency [interquartile range]. Statistics: The AMR values [interquartile range] were obtained using a GALMSS with zero-one inflated beta distribution and multivariate analysis was performed with age, sex, and antiretroviral therapy by stepwise. **a**: comparison between LLV and NHC groups; **b**: comparison between SV and NHC groups; **c**: comparison between LLV and SV groups. Abbreviations: LLV, PWH with persistent low-level viremia (50-200 copies/mL); SV: PWH with suppressed viremia (<50 copies/mL); NHC; non-HIV controls; aAMR, adjusted Arithmetic Median Ratio; q, corrected level of significance by false discovery rate; N, naïve T-cells; CM, central memory T cells; EM, memory effector T-cells, TEMRA pE1, pre-terminally differentiated effector memory RA+ 1 T cells; TEMRA pE2, intermediate differentiated effector memory RA+ 2 T cells; TEMRA E, terminally differentiated effector memory RA+ T cells; HAVCR2 also known as TIM3.

# **Supplementary Material 11. Comparison of systemic inflammation between all groups of study.**

|  | **LLV** | **SV** | **NHC** | **aAMR^a^**  **[IC95%]** | **p^a^** | **q^a^** | **aAMR^b^**  **[IC95%]** | **p^b^** | **q^b^** | **aAMR^c^**  **[IC95%]** | **p^c^** | **q^c^** |
| --- | --- | --- | --- | --- | --- | --- | --- | --- | --- | --- | --- | --- |
| **Th1/Th2** | | | | | | | | | | | | |
| GM-CSF | 18.50 [14.75-21.50] | 17.00 [13.00-20.75] | 15.25 [11.00-20.75] | 1.29 [0.91-1.81] | 0.153 | 0.376 | 1.14 [0.86-1.52] | 0.358 | 0.597 | 1.12 [0.83-1.51] | 0.467 | 0.948 |
| IFN-γ | 160.50 [124.75-194.00] | 124.00 [97.25-175.50] | 23.00 [19.25-30.00] | 5.43 [4.01-7.36] | **<0.001** | **<0.001** | 4.96 [3.57-6.89] | **<0.001** | **<0.001** | 1.11 [0.89-1.39] | 0.368 | 0.948 |
| IL1-β | 23.00 [18.50-26.00] | 23.00 [19.00-31.50] | 18.00 [11.75-21.50] | 1.02 [0.73-1.44] | 0.901 | 0.943 | 1.15 [0.84-1.57] | 0.401 | 0.607 | 0.91 [0.78-1.07] | 0.250 | 0.948 |
| IL-2 | 18.00 [15.25-21.00] | 16.00 [14.00-21.50] | 16.00 [13.25-21.00] | 1.00 [0.58-1.72] | 0.988 | 0.998 | 0.90 [0.59-1.39] | 0.644 | 0.874 | 1.06 [0.74-1.50] | 0.767 | 0.948 |
| IL-4 | 27.00 [23.50-31.00] | 28.00 [23.50-31.00] | 17.50 [13.00-23.75] | 1.46 [1.12-1.89] | **0.007** | **0.026** | 1.47 [1.15-1.88] | **0.003** | **0.014** | 0.98 [0.79-1.20] | 0.819 | 0.948 |
| IL-5 | 22.00 [20.25-27.00] | 20.00 [18.00-24.50] | 19.00 [13.00-26.25] | 1.07 [0.82-1.38] | 0.630 | 0.777 | 1.02 [0.79-1.32] | 0.872 | 0.957 | 1.05 [0.88-1.26] | 0.578 | 0.948 |
| IL-6 | 16.50 [15.50-21.50] | 18.00 [13.75-24.25] | 16.50 [12.00-23.00] | 1.10 [0.75-1.61] | 0.640 | 0.777 | 1.17 [0.83-1.64] | 0.373 | 0.600 | 0.95 [0.63-1.43] | 0.806 | 0.948 |
| IL-8 | 92.00 [82.12-158.25] | 102.00 [76.00-126.00] | 62.50 [51.75-75.50] | 1.68 [1.30-2.18] | **<0.001** | **0.002** | 1.82 [1.30-2.55] | **0.001** | **0.005** | 0.93 [0.66-1.30] | 0.658 | 0.948 |
| IL-12 | 23.00 [19.00-25.50] | 21.25 [17.12-29.38] | 19.00 [15.25-22.75] | 1.06 [0.84-1.33] | 0.652 | 0.777 | 1.03 [0.82-1.30] | 0.774 | 0.942 | 1.01 [0.86-1.19] | 0.885 | 0.948 |
| IL-13 | 24.50 [16.75-28.75] | 21.25 [17.00-24.75] | 14.50 [10.75-16.50] | 1.49 [1.10-2.03] | **0.014** | **0.045** | 1.28 [0.98-1.66] | 0.076 | 0.222 | 1.18 [0.93-1.49] | 0.187 | 0.948 |
| IL-18 | 745.00 [599.50-1039.75] | 652.00 [421.50-836.25] | 67.00 [48.00-92.50] | 10.72 [8.45-13.60] | **<0.001** | **<0.001** | 10.20 [7.47-13.92] | **<0.001** | **<0.001** | 0.91 [0.66-1.25] | 0.567 | 0.948 |
| TNF-α | 30.50 [25.75-34.00] | 29.00 [25.00-33.25] | 15.00 [12.50-20.50] | 1.64 [1.24-2.15] | **0.001** | **0.005** | 1.77 [1.37-2.30] | **<0.001** | **<0.001** | 0.99 [0.82-1.21] | 0.939 | 0.961 |
| **Th9/Th17/Th22/Treg** | | | | | | | | | | | | |
| IL-9 | 28.50 [24.25-34.00] | 26.50 [21.75-31.50] | 27.00 [22.50-38.75] | 0.96 [0.67-1.36] | 0.804 | 0.905 | 0.85 [0.66-1.09] | 0.204 | 0.417 | 1.18 [0.87-1.62] | 0.297 | 0.948 |
| IL-10 | 22.00 [17.00-25.50] | 18.50 [13.25-26.50] | 17.50 [16.00-22.25] | 1.13 [0.90-1.43] | 0.296 | 0.506 | 1.02 [0.77-1.36] | 0.880 | 0.957 | 1.10 [0.86-1.41] | 0.449 | 0.948 |
| IL-17A | 21.00 [17.00-26.00] | 16.00 [13.75-26.00] | 18.50 [13.75-23.50] | 1.17 [0.73-1.86] | 0.514 | 0.747 | 0.99 [0.69-1.42] | 0.959 | 0.981 | 1.11 [0.74-1.66] | 0.607 | 0.948 |
| IL-21 | 30.00 [24.25-72.50] | 30.00 [20.50-36.00] | 31.50 [22.00-57.50] | 1.70 [0.78-3.69] | 0.188 | 0.404 | 2.01 [0.83-4.90] | 0.129 | 0.324 | 0.86 [0.34-2.13] | 0.738 | 0.948 |
| IL-22 | 49.00 [33.00-123.75] | 35.50 [29.50-60.00] | 65.00 [35.50-82.00] | 1.33 [0.59-3.04] | 0.495 | 0.742 | 1.78 [0.70-4.51] | 0.230 | 0.443 | 0.75 [0.27-2.05] | 0.579 | 0.948 |
| IL-23 | 22.25 [18.25-28.00] | 20.00 [16.50-26.00] | 17.50 [14.00-26.25] | 1.22 [0.57-2.60] | 0.611 | 0.777 | 1.35 [0.83-2.22] | 0.236 | 0.443 | 1.23 [0.69-2.20] | 0.479 | 0.948 |
| IL-27 | 20.00 [14.50-24.75] | 17.00 [11.50-21.50] | 15.00 [12.75-23.75] | 1.36 [0.87-2.13] | 0.189 | 0.404 | 0.99 [0.69-1.41] | 0.938 | 0.981 | 1.38 [0.88-2.15] | 0.163 | 0.948 |
| **Inflammatory cytokines** | | | | | | | | | | | | |
| INF-α | 32.00 [24.50-35.25] | 30.00 [23.75-36.50] | 29.50 [24.25-51.00] | 1.10 [0.71-1.72] | 0.665 | 0.777 | 1.04 [0.67-1.61] | 0.864 | 0.957 | 1.06 [0.62-1.83] | 0.830 | 0.948 |
| IL-1α | 42.00 [36.75-75.75] | 42.00 [34.50-60.75] | 40.00 [34.75-62.50] | 1.48 [0.76-2.88] | 0.249 | 0.487 | 1.25 [0.74-2.09] | 0.405 | 0.607 | 1.10 [0.58-2.09] | 0.767 | 0.948 |
| IL-1RA | 254.50 [183.75-449.25] | 217.00 [143.50-386.25] | 31.50 [20.00-35.00] | 10.70 [7.31-15.65] | **<0.001** | **<0.001** | 9.67 [6.41-14.57] | **<0.001** | **<0.001** | 1.08 [0.76-1.55] | 0.665 | 0.948 |
| IL-7 | 21.25 [17.12-25.00] | 22.00 [18.75-25.50] | 17.50 [15.00-26.25] | 0.99 [0.77-1.28] | 0.966 | 0.988 | 1.02 [0.80-1.30] | 0.893 | 0.957 | 0.98 [0.85-1.14] | 0.825 | 0.948 |
| IL-15 | 24.00 [19.50-30.50] | 24.00 [19.00-30.50] | 22.00 [19.00-31.00] | 1.09 [0.75-1.59] | 0.643 | 0.777 | 0.95 [0.73-1.23] | 0.680 | 0.874 | 1.12 [0.84-1.49] | 0.445 | 0.948 |
| IL-31 | 29.50 [26.12-33.75] | 28.00 [24.75-32.25] | 27.00 [23.50-32.25] | 1.13 [0.90-1.43] | 0.304 | 0.506 | 1.04 [0.88-1.21] | 0.673 | 0.874 | 1.12 [0.92-1.36] | 0.276 | 0.948 |
| LTα | 52.00 [45.00-58.75] | 50.00 [43.00-60.00] | 50.00 [41.50-56.25] | 1.10 [0.80-1.52] | 0.563 | 0.777 | 0.93 [0.72-1.21] | 0.606 | 0.852 | 1.18 [0.90-1.55] | 0.243 | 0.948 |
| **Chemokines** | | | | | | | | | | | | |
| Eotaxin | 253.00 [137.25-391.00] | 296.50 [160.00-423.50] | 98.00 [78.00-158.00] | 2.77 [1.91-4.02] | **<0.001** | **<0.001** | 3.12 [2.03-4.79] | **<0.001** | **<0.001** | 0.90 [0.57-1.43] | 0.655 | 0.948 |
| Gro-α | 48.50 [38.50-63.88] | 48.00 [38.00-58.00] | 38.00 [34.25-52.25] | 1.25 [0.98-1.58] | 0.075 | 0.226 | 1.22 [0.91-1.64] | 0.180 | 0.385 | 0.90 [0.69-1.16] | 0.411 | 0.948 |
| CXCL10 | 177.00 [126.50-221.25] | 129.00 [106.75-252.75] | 109.00 [87.25-154.00] | 1.51 [1.15-1.98] | **0.005** | **0.020** | 1.55 [1.08-2.22] | **0.021** | **0.074** | 0.97 [0.69-1.38] | 0.881 | 0.948 |
| CCL2 | 233.00 [190.75-294.25] | 163.50 [102.75-467.25] | 161.00 [107.00-267.75] | 1.22 [0.85-1.77] | 0.287 | 0.506 | 1.24 [0.83-1.87] | 0.300 | 0.520 | 0.75 [0.46-1.21] | 0.245 | 0.948 |
| CCL3 | 50.25 [35.12-74.75] | 47.00 [34.75-75.00] | 38.50 [30.00-67.50] | 1.10 [0.71-1.72] | 0.674 | 0.777 | 1.04 [0.66-1.63] | 0.866 | 0.957 | 1.06 [0.68-1.64] | 0.799 | 0.948 |
| CCL4 | 175.00 [111.50-307.75] | 181.50 [134.25-332.50] | 137.50 [99.50-190.50] | 1.42 [0.88-2.30] | 0.155 | 0.376 | 1.50 [0.93-2.43] | 0.106 | 0.280 | 0.83 [0.50-1.37] | 0.460 | 0.948 |
| CCL5 | 6663.50 [5530.38-7681.00] | 6589.00 [5266.75-7019.00] | 5365.00 [4275.00-6217.00] | 1.23 [1.07-1.41] | **0.005** | **0.021** | 1.19 [1.04-1.37] | **0.015** | **0.054** | 1.03 [0.92-1.15] | 0.636 | 0.948 |
| CXCL12 | 258.00 [164.75-376.75] | 226.00 [183.50-275.50] | 199.50 [154.00-317.00] | 1.16 [0.79-1.71] | 0.460 | 0.735 | 1.19 [0.78-1.81] | 0.426 | 0.619 | 0.98 [0.63-1.52] | 0.922 | 0.961 |
| **Growth factors** | | | | | | | | | | | | |
| BDNF | 1986.00 [1126.00-3194.50] | 2050.50 [1211.75-3187.75] | 557.00 [410.00-994.50] | 3.12 [2.19-4.44] | <**0.001** | **<0.001** | 3.35 [2.36-4.77] | **<0.001** | **<0.001** | 0.93 [0.65-1.32] | 0.690 | 0.948 |
| EGF | 47.00 [36.50-96.50] | 50.50 [33.50-88.00] | 26.00 [22.00-49.00] | 1.74 [1.21-2.50] | **0.005** | **0.020** | 1.67 [1.17-2.37] | **0.007** | **0.027** | 1.03 [0.73-1.44] | 0.879 | 0.948 |
| FGF-2 | 42.00 [34.50-65.00] | 40.00 [34.50-57.75] | 35.50 [29.00-46.50] | 0.97 [0.67-1.41] | 0.883 | 0.943 | 0.94 [0.65-1.36] | 0.755 | 0.942 | 1.03 [0.82-1.30] | 0.794 | 0.948 |
| HGF | 29.00 [22.25-31.25] | 27.00 [24.75-33.25] | 20.00 [16.50-26.50] | 1.19 [0.94-1.52] | 0.159 | 0.376 | 1.27 [0.98-1.65] | 0.079 | 0.222 | 0.95 [0.81-1.11] | 0.499 | 0.948 |
| LIF | 23.00 [17.25-25.00] | 18.00 [15.75-21.25] | 17.00 [13.50-23.00] | 1.25 [0.84-1.84] | 0.272 | 0.506 | 1.00 [0.76-1.32] | 0.990 | 0.990 | 1.20 [0.89-1.62] | 0.232 | 0.948 |
| NGF-β | 27.50 [24.25-34.00] | 26.50 [24.25-29.50] | 28.00 [22.25-31.75] | 1.09 [0.86-1.38] | 0.474 | 0.735 | 1.28 [0.91-1.79] | 0.160 | 0.370 | 0.90 [0.61-1.33] | 0.599 | 0.948 |
| PDGF-BB | 274.00 [104.00-446.00] | 352.50 [212.50-419.00] | 119.00 [93.50-231.25] | 2.44 [1.35-4.43] | **0.005** | **0.020** | 2.29 [1.54-3.39] | **<0.001** | **0.001** | 1.00 [0.57-1.78] | 0.987 | 0.948 |
| PIGF-1 | 240.00 [161.75-329.25] | 234.00 [193.50-310.00] | 196.00 [162.00-246.00] | 1.21 [0.90-1.63] | 0.206 | 0.421 | 1.25 [1.00-1.55] | 0.057 | 0.183 | 1.03 [0.79-1.35] | 0.813 | 0.948 |
| KITLG | 66.00 [54.75-94.50] | 69.00 [57.00-101.00] | 70.00 [49.00-97.00] | 1.02 [0.80-1.30] | 0.871 | 0.943 | 1.16 [0.88-1.52] | 0.300 | 0.520 | 0.88 [0.67-1.15] | 0.339 | 0.948 |
| VEGF-A | 100.00 [67.00-197.00] | 116.00 [83.75-157.25] | 44.00 [33.50-70.00] | 2.70 [1.72-4.24] | **<0.001** | **0.001** | 2.43 [1.49-3.94] | **0.001** | **0.004** | 0.95 [0.54-1.67] | 0.855 | 0.948 |
| VEGF-D | 43.00 [36.50-65.00] | 51.00 [35.75-56.50] | 39.00 [32.25-50.00] | 1.19 [0.97-1.47] | 0.106 | 0.297 | 1.17 [0.94-1.46] | 0.164 | 0.370 | 0.93 [0.74-1.18] | 0.567 | 0.948 |

**Note**. Values are expressed as median of fluorescence [interquartile range]. Statistics: The AMR values [interquartile range] were obtained using generalized linear model with a gamma distribution and multivariate analysis was performed with age, sex, and antiretroviral therapy by stepwise. **a**: comparison between LLV and NHC groups; **b**: comparison between SV and NHC groups; **c**: comparison between LLV and SV groups. Abbreviations: LLV, PWH with persistent low-level viremia (50-200 copies/mL); SV: PWH with suppressed viremia (<50 copies/mL); NHC; non-HIV controls; aAMR, adjusted Arithmetic Median Ratio; q, corrected level of significance by false discovery rate, LTα, also known as TNF-β.
